# Factors that predict life sciences student persistence in undergraduate research experiences

**DOI:** 10.1371/journal.pone.0220186

**Published:** 2019-08-14

**Authors:** Katelyn M. Cooper, Logan E. Gin, Barierane Akeeh, Carolyn E. Clark, Joshua S. Hunter, Travis B. Roderick, Deanna B. Elliott, Luis A. Gutierrez, Rebecca M. Mello, Leilani D. Pfeiffer, Rachel A. Scott, Denisse Arellano, Diana Ramirez, Emma M. Valdez, Cindy Vargas, Kimberly Velarde, Yi Zheng, Sara E. Brownell

**Affiliations:** 1 The Biology Education Research Lab, Research for Inclusive STEM Education Center, School of Life Sciences, Arizona State University, Tempe, Arizona, United States of America; 2 LEAP Scholars, Research for Inclusive STEM Education Center, Arizona State University, Tempe, Arizona, United States of America; 3 Mary Lou Fulton Teachers College, Arizona State University, Tempe, Arizona, United States of America; Indiana University, UNITED STATES

## Abstract

Undergraduate research experiences (UREs) have the potential to benefit undergraduates and longer UREs have been shown to lead to greater benefits for students. However, no studies have examined what causes students to stay in or consider leaving their UREs. In this study, we examined what factors cause students to stay in their UREs, what factors cause students to consider leaving their UREs, and what factors cause students to leave their UREs. We sampled from 25 research-intensive (R1) public universities across the United States and surveyed 768 life sciences undergraduates who were currently participating in or had previously participated in a URE. Students answered closed-ended and open-ended questions about factors that they perceived influenced their persistence in UREs. We used logistic regression to explore to what extent student demographics predicted what factors influenced students to stay in or consider leaving their UREs. We applied open-coding methods to probe the student-reported reasons why students chose to stay in and leave their UREs. Fifty percent of survey respondents considered leaving their URE, and 53.1% of those students actually left their URE. Students who reported having a positive lab environment and students who indicated enjoying their everyday research tasks were more likely to not consider leaving their UREs. In contrast, students who reported a negative lab environment or that they were not gaining important knowledge or skills were more likely to leave their UREs. Further, we identified that gender, race/ethnicity, college generation status, and GPA predicted which factors influenced students’ decisions to persist in their UREs. This research provides important insight into how research mentors can create UREs that undergraduates are willing and able to participate in for as long as possible.

## Introduction

Undergraduate research has been championed as a high-impact educational practice that results in significant benefits for students who participate [1–5]. Prior studies have demonstrated that engaging in undergraduate research experiences (UREs) can increase student perceived understanding of how to conduct a research project, as well as student confidence in research skills [6,7]. Further, UREs can enhance students’ abilities to think critically [8–10] and improve student learning [9,11,12]. Importantly, participating in research has been shown to positively influence student persistence in undergraduate science, technology, engineering, and math (STEM) degrees [13–15], as well as students’ interests in pursuing STEM graduate programs and their chances of being accepted to such programs [6–8,13,16–21].

To maximize the benefits that students could gain from UREs, it is imperative to identify what specific aspects of undergraduate research lead to such positive outcomes [22]. One factor well known to maximize a student’s chance of reaping research benefits is the duration of a student’s URE [6,23–27]. Longer research experiences provide undergraduates with the time needed to develop the thought processes, skills, and relationships that can be leveraged into personal and professional gains. Specifically, compared to undergraduates in their first year of research, students who had participated in a multi-year URE reported greater gains in research skills, such as analyzing data, problem solving, and identifying flaws in the interpretation of data [26]. A study conducted at a Hispanic-serving institution came to similar conclusions; students who participated in research longer were more likely to report greater gains in thinking and working like a scientist (e.g. analyzing data, problem solving, formulating research questions), as well as greater personal gains (e.g. confidence in one’s ability to contribute to science, comfort working with others, and the ability to work independently) [24]. Additionally, a study examining undergraduates’ perceived gains as a result of time spent in a single URE found that participants reported more gains, including increased confidence in research skills and in their ability to succeed in graduate school, at the end of the yearlong experience than they did at the midpoint of their experience [23]. Notably, faculty mentors also perceive that undergraduates who have longer UREs benefit more. A survey of science and engineering faculty found that faculty members who supervised undergraduates for two or more years believed that the impact of research on students’ skills, such as developing intellectual curiosity, solving problems independently, and approaching problems creatively, was significantly greater than faculty members who typically supervised undergraduate researchers for a year or less [27]. Finally, longer UREs have also been correlated with an increased likelihood of pursuing a career in STEM and have been found to be a robust predictor of student research performance in STEM Ph.D. programs [6,28].

Lengthier UREs do not only benefit students; undergraduates who spend a longer time in a single lab can also positively impact research mentors [29,30]. Research mentors, including faculty, postdocs, and graduate students, spend a significant amount of time and effort training undergraduates so that the student can benefit from the research experience, but also so that the student can become a productive member of the lab [31–34]. Faculty members have highlighted the steep cost of training undergraduates; choosing to train an undergraduate may result in less research productivity than if the faculty member had chosen to spend that time training a graduate student who would likely stay in the lab for longer and be more productive [25]. Therefore, it is unsurprising that faculty members cite the turnover of undergraduate researchers as a significant cost of taking undergraduates into their labs [32]. Faculty mentors agree that undergraduates can contribute to faculty research goals to publish or present work, but that this does not typically happen until the undergraduate has been in the lab for an extended period of time [29,32]. In fact, a recent study demonstrated that faculty mentors were more productive in publishing collaboratively with undergraduate students when they worked with students for more than one year [30].

The well-established student benefits of longer research experiences have resulted in calls for undergraduates to participate in research earlier in their academic careers [5,22,27,35]. Importantly, this recommendation is often predicated on the assumption that once a student joins a research lab, they will persist in their research experience until the end of their undergraduate career. Yet, some students choose to leave their URE before graduating and many faculty members have had a student join their lab and not persist until graduation. However, we know very little about what causes students to leave research experiences prematurely. Some studies have pinpointed factors that may cause students to leave their UREs by identifying student frustrations and difficulties regarding their UREs [7,36]. Specifically, Seymour and colleagues found that students became frustrated in their research when they were not gaining specific research skills [7], and Mabrouk and Peters identified student difficulties in research including differences between faculty and student perceptions of the necessary time commitment for research, balancing competing demands of classes, work, and research, inadequate support from research mentors, self-motivation, and student fear of failure [36]. A study exploring the experiences of underrepresented students in UREs concluded that the absence of constructive feedback could cause students to leave their UREs, especially if students are afraid of making a mistake. Additionally, Thiry and colleagues identified that some students’ negative research experiences caused them to change their career and educational plans, and that these students were often given little or no direction regarding their research activities [37]. While these studies shed light on what factors may influence students’ decisions to leave their research experiences, to our knowledge, no studies have systematically explored what contributes to whether a student chooses to leave their URE.

Identifying what influences students to persist in their UREs as well as what causes them to leave prematurely could provide unique insight into how research mentors can create research environments where undergraduates are willing and able to participate for as long as possible. Further, exploring whether the factors that cause students to consider leaving their UREs vary across students in different demographic groups may shed light on why certain demographic groups are underrepresented in science careers. While studies suggest that undergraduate researchers are demographically diverse, with the reported percentage of female students, Black students, and Latino/a students in research being greater than or equal to the percent in the overall college population [6], there are still inequities with regard to who pursues a STEM Ph.D. Specifically, female students, Black students, and Latino/a students are less likely to obtain a science or engineering Ph.D. than their male and white counterparts [38]. Students with similar interests in STEM careers may have research experiences differing in quality and duration in part because of their demographics or backgrounds, which may ultimately lead these students to disproportionately choose not to pursue STEM careers. While studies have highlighted gender differences with regard to students’ preferences and perceptions of undergraduate research [36,39], to our knowledge, no studies have identified whether student demographics predict what factors influence student decisions to leave their UREs before graduating.

Theories that aim to explain human motivation provide useful frameworks for examining students’ decisions to stay in or leave their UREs. For example, expectancy value theory [40] suggests that in order to complete a task, one must believe they would be successful at the task, and must weigh the value and the cost of completing the task [41–43]. Applied to UREs, this would suggest that a student who chooses to leave their URE may not believe they are good at research, may not value participating in research, or may perceive that the costs associated with staying in their URE are too high. Another relevant theory of human motivation is Maslow’s hierarchy of needs, which suggests that people will first aim to fulfill basic needs that are unmet, such as financial stability [44]. Only once those needs are met will they seek to fulfill social needs, such as feeling a sense of belonging and establishing friendships, followed by esteem needs, such as prestige and respect from others, and finally self-actualization needs, such as seeking personal growth. This theory suggests that undergraduates may differ in what motivates them to stay in their URE depending on which of their needs are currently being met [44]. We do not expect that any one motivational theory will fully explain the complex decisions that students make to stay in or leave their UREs. However, these theories may provide useful insight into some aspects of students’ decision-making.

In this study, we aimed to document the reasons why students choose to stay in or leave their first URE by surveying a national population of undergraduates who are currently participating in, or had previously participated in, an academic year URE. We used a mixed-methods approach with the intent to systematically explore to what extent there are demographic differences in students’ reasonings while allowing for a deeper qualitative exploration of students’ decisions to stay in or leave their UREs.

## Methods

This study was done with an approved Arizona State University Institutional Review Board protocol #7247. All participants are adults and provided written consent to participate in the study at the beginning of the survey.

This research project was conducted as part of a four-semester-long science education course-based undergraduate research experience (CURE) taught by K.M.C., L.E.G., and S.E.B. in fall 2017, spring 2018, fall 2018, and spring 2019. In a CURE, undergraduates engage in novel, broadly relevant research in the context of a formal course [45–48]. This course was backward designed to enhance students’ scientific thinking skills and understanding of undergraduate research in order to prepare students to enter basic science research experiences in a subsequent semester [49]. Fourteen students were enrolled in the CURE and were researchers on this project. Collectively, the instructors of the course and the student researchers were responsible for developing the research questions, collecting the data, analyzing the data, interpreting the data, and communicating the findings.

### Survey design and distribution

In fall 2017, we designed an open-ended pilot survey probing what causes students to stay in, consider leaving, and actually leave their first UREs. The survey was sent to 3723 life sciences students at a large public research-intensive (R1) institution in the Southwest, and 126 students who were participating in a URE or who had previously participated in a URE completed the survey. We analyzed the data collected in the fall 2017 pilot survey with the intention of using the results to inform a revised survey in 2018.

In fall 2018, we developed a new survey based on the 2017 pilot data to assess why students stay in, consider leaving, and actually leave their first UREs. Specifically, the open-ended survey questions were revised, and we developed additional closed-ended questions based on student responses to the open-ended questions in the fall 2017 survey. Seventeen of the researchers iteratively reviewed the survey questions using a set of criteria that we developed to assess the appropriateness of each question (e.g. Is the question grammatically correct? Is the meaning and interpretation of the question clear? Are the question answer choices unambiguous in meaning?) [50]. The researchers provided written feedback about each question and the survey was revised. Next, seven of the researchers conducted a series of think-aloud interviews with a total of 14 undergraduate life sciences students who had done research to establish cognitive validity of the survey by ensuring that students understood what each question was asking. The survey was iteratively revised after the think-aloud interviews [51]. Seventeen of the researchers completed the revised survey and again evaluated each question using the criteria for assessing survey questions. The survey was revised based on their feedback to create the final 2018 survey. All references to the survey from here forward refer to the revised 2018 survey; please see the S1 File for a copy of the final survey questions that were administered to students.

The survey was deployed using the online platform Qualtrics. The survey was sent to a national sample of undergraduate life sciences students. After identifying “R1- highest research activity” public institutions based on the Carnegie classifications [52], we used university websites to identify an individual from each institution who likely had access to an email listserv of students in their life sciences department (e.g. manager of undergraduate programs, undergraduate program coordinator) and contacted each individual via email. We sent one follow up email to any institution that did not respond to our initial request. We chose to recruit exclusively from public R1 institutions to control for any potential differences among the experiences of students in undergraduate research that may exist across different institution types. For example, public R1 institutions typically have a larger student population and the ratio of students to research positions makes obtaining a research position typically more competitive than at a private R1 institution [53]. We were also purposeful in our sampling approach in that we did not target students who were enrolled in established research programs or students presenting at conferences because we wanted our sample to be as representative as possible of a typical undergraduate researcher. Because a student’s college major can impact what a student gains from their research experience [54], we limited our study to life sciences majors. Of the 81 R1 institutions that were contacted, 25 institutions (31%) agreed to send out the survey to undergraduate students in their life sciences department. Students were offered a chance to win a $50 gift card for completing the survey. Eight-hundred students completed the survey and of those students, 768 were majoring in the life sciences. The number of life sciences students who responded to the survey from each anonymized institution can be found in the S1 File.

### Students’ experiences in undergraduate research

The survey asked students a series of questions about their first URE during the academic year, as well as a set of demographic questions. First, students were asked whether they were currently participating or had previously participated in a scientific undergraduate research experience, defined as working with a faculty member or in a faculty member’s lab while enrolled in college. Students were then asked to specify if they had only participated in a summer research experience that they did not continue during the academic school year (e.g. an NSF Research Experience for Undergraduates program). The purpose of these questions was to invite only students who had participated in undergraduate research during the academic school year to complete the survey. We were only interested in surveying students who had participated in academic year research experiences because our questions about choosing to leave one’s URE were not applicable to students who had only participated in a summer research program with a specific endpoint.

**Fig 1:**
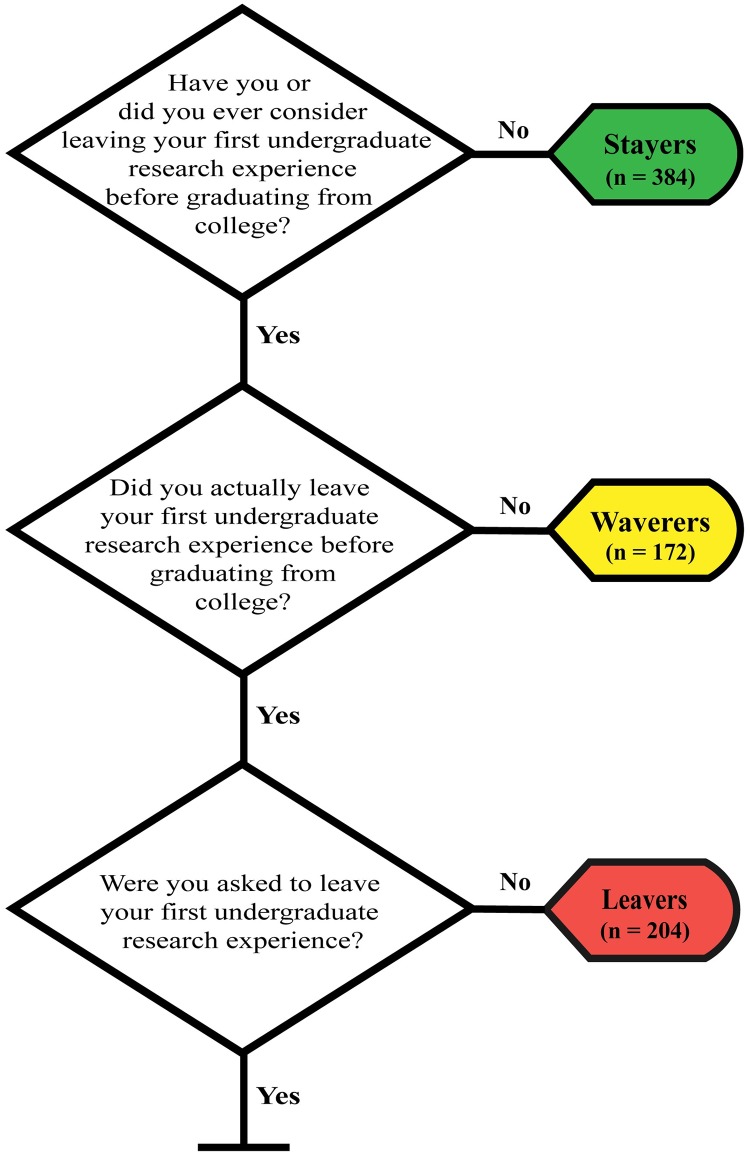
Flow chart of the survey questions that were used to determine whether students were considered stayers, waverers, or leavers and the number of survey participants who were classified as each group.

Students who had participated in academic year undergraduate research were asked a series of questions about whether they had ever considered leaving or actually left their first URE. The flowchart of these questions is displayed in Fig 1. Students who did not consider leaving their first research experience will be referred to as **stayers**, students who considered leaving their research experience but ultimately stayed will be referred to as **waverers**, and students who left their research experience will be referred to as **leavers**. All students who chose to stay in their first URE (stayers and waverers) were asked an open-ended question about why they chose to stay. After they completed the open-ended question, they were presented with a list of 11 factors that may have influenced their choice to stay in their URE, which were developed from student responses to the 2017 pilot survey. Students were asked to select all factors that caused them to stay in their URE or to skip the question if none of the factors applied to them. The purpose of this closed-ended question was to gain a more holistic understanding of all of the possible reasons why students chose to stay in research, compared to the more salient reasons that we expected would emerge from the open-ended question. Similarly, all students who either considered leaving their first URE but ultimately stayed (waverers) and students who considered leaving their first URE and left (leavers) were given an open-ended question asking them why they considered leaving. After completing the open-ended question, students were presented with 11 factors that may have caused them to consider leaving their URE, which were also developed from student responses to the pilot survey. Students were asked to select all factors that caused them to consider leaving their first URE, or to skip the question if none of the factors applied to them. The small number of students who reported being asked to leave their first URE were not included in any analyses.

### Analysis of the extent to which demographics predict students’ thoughts and behaviors regarding their URE

We used logistic regression to test to what extent demographics predicted students’ thoughts and behaviors regarding their first UREs. Specifically, we explored gender (male, female), race/ethnicity (underrepresented racial minority (URM), Asian, white), college generation status (first generation, continuing generation), and grade point average (GPA) as predictor variables. We recognize that not all students identify as gender binary (male or female) however, there were too few students who identified as a gender other than male or female to create a third category. We collapsed students who identify as Black or African American, Hispanic, Latino/a or of Spanish Origin, American Indian or Alaska Native, and Pacific Islander into one category, which we call underrepresented racial minority (URM) students. These students share the experience of being underserved by institutions of higher education; we recognize that the experiences of these students are different, but the small sample sizes necessitated that we pool these identities as a single factor in our analyses. We chose not to nest students within institutions in any of our analyses because we had no reason to think that the experience of any two students in different labs at a single institution would be the same, and thus would be fundamentally different from the experience of students at another institution. While there may be similarities between the experiences of students in the same lab, we were not able to control for this as we did not collect data identifying their research lab to help protect the identity of the students and to encourage them to share honestly about their experiences. We tested to what extent student demographics predicted: A. whether students who stayed in their first URE considered leaving (model: stayer/waverer ~ gender + race/ethnicity + college generation status + GPA), and B. whether students who considered leaving their first URE actually left (model: waverer/leaver ~ gender + race/ethnicity + college generation status + GPA).

### Coding and analysis of students’ responses to open-ended survey questions about why they chose to stay in or considered leaving their URE

Seventeen researchers reviewed all student responses to the open-ended question asking students why they chose to stay in their URE. The researchers used inductive coding and took notes as they independently coded a subset of student responses in order to identify themes that emerged from the data [55]. All of the researchers then compared their themes, agreed on a common set of themes, and developed a rubric describing each theme. A team of five researchers (K.M.C., L.E.G., B.A., J.S.H., and T.B.R.) finalized the rubric using constant comparative methods. Excerpts of student responses about why they stayed in research were continuously compared to ensure that each theme adequately represented the respective quotes and that quotes were not so different from one another as to warrant the creation of a new theme [56]. Next, each of the five researchers coded all responses to the question independently and then met together to review discrepancies about any codes and discuss differences until coming to consensus [57]. This process was repeated for the open-ended question about why students considered leaving their URE. Within a student’s response, a single phrase could only be coded as one theme, however students’ responses often included more than one theme. Descriptions of the themes identified for both questions are included in the S1 File.

### Analysis of students’ responses to closed-ended survey questions about why they chose to stay in or considered leaving their URE

To analyze the closed-ended question asking students to select any of the 11 factors that influenced their choice to stay in their URE, we used logistic regression to test to what extent student demographics (gender, race/ethnicity, college generation status, and GPA) predicted whether a student would select a particular factor that influenced their decision to stay in their URE (model: whether student selected a particular factor ~ gender + race/ethnicity + college generation status + GPA). We used chi square tests of independence to determine whether there were differences in the percent of stayers and waverers who selected each factor. We also used logistic regression to test whether selecting any of the 11 factors predicted whether a student did not consider leaving or considered leaving but ultimately stayed in research (model: stayer/waverer ~ reason A + reason B + reason C + …).

Similarly, to analyze the closed-ended question asking students to select any of the 11 factors that influenced them to consider leaving their URE, we used logistic regression to test to what extent student demographics (gender, race/ethnicity, college generation status, and GPA) predicted whether a student would select a particular factor that influenced them to consider leaving (model: whether student checked a particular factor ~ gender + race/ethnicity + college generation status + GPA). We used chi square tests of independence to test whether there were differences in the percent of waverers and leavers who selected each factor. We used logistic regression to test whether selecting any of the 11 factors predicted whether a student considered leaving but stayed or left their URE (model: waverer/leaver ~ reason A + reason B + reason C + …).

We recognize that the significance of a result from any statistical test is continuous rather than dichotomous based on the specific p-value [58]. However, we report select results by the criterion of p ≤ 0.05 throughout the results section for simplicity. We acknowledge that test results with p-values greater than 0.05 can still be scientifically meaningful, thus we report out all results of statistical tests in the S1 File for the reader’s further interpretation.

## Results

### Finding 1: Half of undergraduate researchers surveyed considered leaving their URE

Of the 768 life sciences students who completed the survey, exactly half of the students (n = 384) had considered leaving their first URE, while the other half had not considered leaving their first research experience. Of the students who considered leaving, 44.8% (n = 172) chose to stay in their first research experience, while 53.1% (n = 204) chose to leave. Additionally, 2.1% of students (n = 8) were asked to leave their first research experience. In sum, 384 students had not considered leaving their first research experience (**stayers)**, 172 students considered leaving their first research experience but ultimately chose to stay (**waverers**), and 204 students considered leaving their first research experience and left (**leavers**).

The demographics of the students in each group are reported in Table 1. When we compared the population of stayers to waverers, we identified that URM students were 2.0 times more likely than white students to not consider leaving their URE (p = 0.04). We did not find any notable demographic differences between waverers and leavers. A table of the results for each test can be found in the S1 File along with additional demographic information including students’ year in college, the average number of hours spent in research per week, and students’ career goals.

**Table 1:** Demographics of undergraduates who did not consider leaving their URE (stayers), who considered leaving their URE but stayed (waverers), and who considered leaving their URE and left (leavers).

	Stayers n = 384 % (n)	Waverers n = 172 % (n)	Leavers n = 204 % (n)
**Student-level demographics**			
Gender			
Female	71.6% (275)	79.6% (137)	74.0% (151)
Male	27.1% (104)	19.2% (33)	22.5% (46)
Other	0.3% (1)	1.2% (2)	1.0% (2)
Decline to state	1.0% (4)	0.0% (0)	2.5% (5)
Race/ethnicity			
Asian	28.1% (108)	25.0% (43)	24.5% (50)
Underrepresented minority (URM)^a^	15.4% (59)	9.3% (16)	14.2% (29)
White	50.8% (195)	59.9% (103)	55.4% (113)
Other^b^	4.2% (16)	3.5% (6)	2.0% (4)
Decline to state	1.6% (6)	2.3% (4)	3.9% (8)
College generation status			
First generation	32.0% (123)	24.4% (42)	27.5% (56)
Continuing generation	66.1% (254)	75.6% (130)	70.1% (143)
Decline to state	1.8% (7)	0.0% (0)	2.5% (5)
Grade Point Average (GPA)			
Mean	3.52	3.59	3.61
Range	2.00–4.00	0.50–4.00	2.00–4.00

^a^Underrepresented minority (URM) includes students who identified as Black or African American, Hispanic, Latino/a, or of Spanish Origin, American Indian or Alaska Native, and Pacific Islander.

^b^Other includes students who identified as “other” and wrote in a race/ethnicity not listed on the survey, such as Middle Eastern or multiracial.

### Finding 2: Students who reported a positive lab environment and students who indicated that they enjoy their everyday research tasks were more likely to not consider leaving their URE

Upon coding students’ responses to the open-ended question about why they chose to stay in their first URE, we found that all themes that were reported by at least 10% of students were reflected in the 11 factors that were provided to students in the closed-ended question (Table 2). Thus, for simplicity, we will primarily report on the analysis of the closed-ended data, although the complete analysis of the open-ended data is reported in the S1 File.

**Table 2:** Percent of students and demographic differences in who selected each factor that influenced their decision to stay in their first URE.

Factor that may have influenced whether a student chose to stay in their first URE	Percent of all students who selected the factor % (n) n = 556	Percent of stayers who selected the factor % (n) n = 384	Percent of waverers who selected the factor % (n) n = 172	Summary of demographic differences among which students reported the factor
I am gaining important skills or knowledge^+^	84.5% (470)	88.3%*** (339)	76.2% (131)	Continuing generation college students were 1.9 times more likely than first generation college students to stay in research because they are gaining important skills or knowledge.
Research experience is important for my future career^+^	84.0% (467)	84.6% (325)	82.6% (142)	Male students were 1.9 times more likely than female students to stay in research because it is important for their career.
The lab is flexible with my schedule/time	82.0% (456)	84.1% (323)	77.3% (133)	
My lab mentor who is a PI, faculty member, graduate student, postdoc or staff member^+^	77.7% (432)	81.8%*** (314)	68.6% (118)	
I am interested in my research topic^+^	73.4% (408)	79.2%*** (304)	60.5% (104)	
The overall environment of my lab^+^	68.9% (383)	78.1%*** (300)	48.3% (83)	
I enjoy my everyday research tasks^+^	57.2% (318)	66.1%*** (254)	37.2% (64)	White students were 2.1 times more likely than URM students to stay in research because they enjoy their everyday research tasks.
I have sufficient guidance for my research project^+^	46.9% (261)	52.6%*** (202)	34.3% (59)	
I have enough time to do research	42.6% (237)	46.6%** (179)	33.7% (58)	
I am concerned I may not have another opportunity	27.3% (152)	20.6% (79)	42.4%*** (73)	Asian students were 1.8 times more likely than white students to stay in research because they are concerned there will not be another opportunity. For every one point decrease in GPA, a student was 1.8 times more likely to select that they are concerned that there will not be another opportunity.
Doing research positively contributes to my financial situation	17.1% (95)	17.7% (68)	15.7% (27)	First generation college students were 1.9 times more likely than continuing generation college students to stay in research because it positively contributes to their financial situation. White students were 2.3 times more likely than Asian students to stay in research because it positively contributes to their financial situation.

Chi-square tests of independence were conducted to test whether there were differences between the percent of stayers and waverers who reported each factor; significant differences are marked with an asterisk:

*p≤0.05,

**p≤0.01,

***p≤0.001.

Logistic regression was used to test to what extent student demographics predicted whether a student would select a particular factor, and the results are summarized in the table.

^+^indicates factors that were reflected by at least 10% of students in the coding of the open-ended data.

Two students (0.4%) did not select any factors.

We tested whether stayers or waverers were more likely to select each of the 11 factors as reasons for staying in their first URE. Additionally, we tested to what extent student demographics predicted whether a student selected a particular factor (Table 2). Stayers checked an average of 7.0 factors, while waverers checked an average of 5.8 factors that contributed to their decision to stay in research. The results tables for all statistical tests can be found in the S1 File.

Before we elaborate on the findings presented in Table 2 above, we would like to first note the technical definition of the language “X.X times more likely” used in the last column of Table 2. This language is a translation of the results of logistic regression. Take the first row of Table 2, “continuing generation college students were 1.9 times more likely than first generation college students to stay in research because they are gaining important skills or knowledge” as an example. The number 1.9 is the natural exponential of the estimated coefficient for the explanatory variable “continuing generation” vs “first generation” in the logistic regression model to predict whether the student reports that “gaining important skills or knowledge” influences their decision to stay in research. This number is also called the “odds ratio,” which is a standardized effect size statistic in logistic regression.

The most frequently selected factor for staying in one’s URE was gaining important skills or knowledge (84.5%). Stayers were more likely to report this factor than waverers (χ^2^ = 13.3, p < 0.001). Continuing generation college students were 1.9 times more likely than first generation college students to select this factor (p = 0.02). The second most selected factor for staying in research was that research is important to the student’s future career (84.0%). Males were 1.9 times more likely than females to report this reason (p = 0.05). The third most common factor influencing why students stayed in research was because the lab was flexible with their time or schedule (82.0%). Students also chose to stay because of their mentor (77.7%), which was more likely to be checked by stayers than waverers (χ^2^ = 11.9, p < 0.001). Here, we use the term “mentor” to refer to anyone, such as a principal investigator (PI), postdoc, or graduate student, who oversees an undergraduate researcher. Over half of the students surveyed chose to stay in research because they were interested in the research topic (73.4%), because of the overall lab environment (68.9%), or because they enjoy their everyday research tasks (57.2%), each of which were more likely to be reported by stayers than waverers (respectively: χ^2^ = 21.3, p < 0.001, χ^2^ = 49.5, p < 0.001, χ^2^ = 40.6, p < 0.001). Additionally, white students were 2.1 times more likely than URM students to report choosing to stay in research because they enjoy their everyday research tasks (p = 0.009). Less than half of students selected having sufficient guidance (46.9%) and having enough time to do research (42.6%) as reasons why they chose to stay; both of which were more likely to be reported by stayers than waverers (respectively: χ^2^ = 16.0, p < 0.001, χ^2^ = 8.1, p < 0.004). Twenty-seven percent of students reported that they chose to stay in their first URE because they were concerned that they may not have another research opportunity, which was more likely to be reported by waverers than stayers (χ^2^ = 28.6, p < 0.001). Further, Asian students were 1.8 times more likely than white students to check this reason (p = 0.01), and for every one-point decrease in a student’s GPA, the student was 1.8 times more likely to select this reason (p = 0.04). Finally, we identified that 17.1% of students stayed in research because doing research contributed positively to their financial situation. First generation college students were 1.9 times more likely than continuing generation college students to report this reason (p = 0.02) and white students were 2.3 times more likely than Asian students to report this reason (p = 0.01).

We also tested to what extent selecting specific factors predicted whether a student would not consider leaving their research experience (stayer) or consider leaving their research experience (waverer). We found that students who reported a positive lab environment were 2.7 times more likely to not consider leaving their research experience than students who did not report a positive lab environment (p < 0.001). Similarly, students who reported enjoying their everyday research tasks were 2.0 times more likely to not consider leaving their first URE than students who did not report enjoying their everyday research tasks (p = 0.003) (Table 3). Given that stayers had not considered leaving, it was unsurprising that students who stayed because they may not have another research opportunity were 2.5 times more likely to be a waverer than a stayer (p < 0.001).

**Table 3:** Results of logistic regression testing to what extent selecting a particular factor predicts whether a student will not consider leaving their first URE.

Model	B ± SE	p-value	Odds ratio
Intercept	-0.05 ± 0.37	0.90	NA
Learning important skills or knowledge	0.26 ± 0.29	0.37	1.30
Research is important for career	-0.13 ± 0.28	0.63	1.14
Lab is flexible with time/schedule	-0.35 ± 0.27	0.20	1.42
Mentor	-0.06 ± 0.26	0.82	1.06
Interested in research topic	0.28 ± 0.24	0.23	1.32
Lab environment	1.00 ± 0.23	<0.001	2.72
Enjoys research tasks	0.67 ± 0.23	0.003	1.95
Has sufficient guidance	0.16 ± 0.23	0.48	1.17
Has enough time for research	0.31 ± 0.22	0.16	1.36
Concerned they may not have another opportunity	-0.91 ± 0.22	<0.001	2.48
Research positively contributes to finances	-0.14 ± 0.28	0.63	1.15

Additional reasons that influenced undergraduates’ decisions to stay in their UREs that were not explicitly asked in the closed-ended questions, but emerged from the open-ended data included: student has independence in the lab (9.0% of students, stayers and waverers combined, reported this), student recognizes personal growth or development in research (8.6%), student has a need to follow through when they commit to something (7.7%), student needs to stay in order to receive an academic benefit (e.g. course credit, complete a thesis) (6.4%), student stays because the lab is accommodating (6.0%), and student hopes to gain a research product, such as a publication (4.8%).

### Finding 3: Students who reported a negative lab environment or who indicated not gaining useful skills or knowledge in their research experience were more likely to leave their URE

Upon coding students’ responses to the open-ended question about why they considered leaving their first URE, we found that all codes that were reported by at least 10% of students were reflected in the 11 factors that were provided to students in the closed-ended question (Table 4). Therefore, we primarily report on the analysis of the closed-ended data. The complete analysis of the open-ended data is reported in the S1 File.

**Table 4:** Percent of students and demographic differences in who selected each factor that influenced them to consider leaving their first URE.

Factor that may have influenced a student to consider leaving their first URE	Percent of all students who selected the factor % (n) n = 376	Percent of waverers who selected the factor % (n) n = 172	Percent of leavers who selected the factor % (n) n = 204	Summary of any demographic differences among which students reported the factor
I did not enjoy my everyday research tasks^+^	43.6% (164)	38.4% (66)	48.0% (98)	Male students were 1.7 times more likely than female students to consider leaving research because they did not enjoy their everyday research tasks. For every one point increase in a student’s GPA, they were 2.3 times more likely to consider leaving because they did not enjoy their everyday research tasks.
I was interested in another research opportunity^+^	39.4% (148)	38.4% (66)	40.2% (82)	
I did not have enough time to do research^+^	38.0% (143)	41.9% (72)	34.8% (71)	For every one point decrease in a student’s GPA, they were 2.1 times more likely to consider leaving because they did not have enough time to do research.
I did not have sufficient guidance for my research project^+^	31.9% (120)	27.3% (47)	35.8% (73)	
My mentor who is a PI, faculty member, postdoc, graduate student, or staff member	30.3% (114)	25.6% (44)	34.3% (70)	
I was not interested in my research topic	29.0% (109)	23.8% (41)	33.3%* (68)	
I needed to spend time making more money than I made doing research	26.9% (101)	26.7% (46)	27.0% (55)	
The overall environment of my lab	26.6% (100)	18.0% (31)	33.8%*** (69)	
I was not gaining important skills or knowledge	18.9% (71)	11.0% (19)	25.5%*** (52)	Underrepresented minority students were 2.6 times more likely than white students to consider leaving research because they were not gaining important skills or knowledge.
Research experience was not important for my future career	14.1% (53)	10.5% (18)	17.2% (35)	Asian students were 2.2 times more likely than white students to consider leaving research because it was not important for their career.
The lab was not flexible with my schedule/time	11.4% (43)	11.0% (19)	11.8% (24)	Female students were 4.0 times more likely than male students to consider leaving research because the lab was not flexible with their time/schedule.

Chi-square tests of independence were conducted to test whether there were differences between the percent of waverers and leavers who reported each factor; significant differences are marked with an asterisk:

*p≤0.05,

**p≤0.01,

***p≤0.001.

Logistic regression was used to test to what extent student demographics predicted whether a student would select a particular factor and the results are summarized in the table.

^+^indicates factors that were reflected by at least 10% of students in the coding of the open-ended data.

Fourteen students (3.7%) did not select any factors.

We tested whether there were differences between the percent of waverers and leavers who checked each factor. Waverers checked an average of 2.4 factors, while leavers checked an average of 3.4 factors. Additionally, we tested to what extent student demographics predicted whether a student selected a particular factor of their research experience that influenced them to consider leaving. The results tables for the regressions can be found in the S1 File.

The most frequently selected factor for considering leaving was that students did not enjoy their everyday research tasks (43.6%) (Table 4). For every one point increase in a student’s GPA, students were 2.3 times more likely to select this reason (p < 0.01). Additionally, male students were 1.7 times more likely than female students to select this factor (p = 0.05). The second most commonly selected factor was students were interested in pursuing another research opportunity (39.4%). Thirty-eight percent of students selected that they did not have enough time to do research, and for every one point decrease in a student’s GPA, they were 2.1 times more likely to select this factor (p = 0.02). Nearly 32% of students checked that they did not have sufficient guidance for their research project, while 30.3% of students identified that they considered leaving or left because of their research mentor. Twenty-nine percent of students selected that they were not interested in the research topic, which was more likely to be checked by leavers than waverers (χ^2^ = 4.1, p = 0.04). Over a quarter of students selected that they needed to spend time making money instead of doing research (26.9%). Additionally, 26.6% of students indicated that the overall environment of the lab influenced them to consider leaving their URE; the overall lab environment was more likely to be selected by leavers than waverers (χ^2^ = 11.9, p < 0.001). Additionally, students considered leaving because they were not gaining important skills or knowledge (18.9%), which was 2.6 times more likely to be reported by URM students than white students (p = 0.02). A subset of students checked that they did not think research was important for their career (14.1%). Asian students were 2.2 times more likely than white students to report that they did not feel research was important for their career (p = 0.04). Finally, 11.4% of students reported that they considered leaving their first URE because the lab was not flexible with their schedule or time; female students were 4.0 times more likely than male students to select this factor (p = 0.03).

We also tested whether selecting specific factors predicted whether a student would actually leave their first URE. We found that students who reported a negative lab environment were 1.6 times more likely to choose to leave their URE than students who did not report a negative lab environment (p = 0.04). Similarly, students who thought they were not gaining important skills or knowledge were 2.1 times more likely to leave their URE than students who did not report this (p = 0.02) (Table 5).

**Table 5:** Results of logistic regression testing whether selecting particular factors predicts whether a student will leave their first URE.

Model	B ± SE	p-value	Odds ratio
Intercept	-0.27± 0.23	0.25	NA
Did not enjoy lab tasks	0.01 ± 0.24	0.92	1.01
Interested in another opportunity	0.07 ± 0.22	0.77	1.07
Not enough time	-0.24 ± 0.23	0.32	1.27
Insufficient guidance	0.07 ± 0.26	0.80	1.07
Mentor	0.02 ± 0.28	0.94	1.02
Not interested in research topic	0.24 ± 0.25	0.34	1.27
Need to make money	0.16 ± 0.25	0.51	1.17
Lab environment	0.62 ± 0.31	0.04	1.86
Not gaining important skills or knowledge	0.72 ± 0.32	0.02	2.05
Research not important for career	0.57 ± 0.33	0.08	1.77
Lab not flexible with time or schedule	-0.02 ± 0.36	0.95	1.02

Additional reasons that influenced undergraduates’ decisions to consider leaving their research experience that were not explicitly asked in the closed-ended questions, but emerged from the open-ended data included: work is tedious or monotonous (6.9% of students, waverers and leavers combined, reported this), lack of personal growth or benefit (6.1%), lack of structure or a disorganized lab (4.5%), feeling overworked or undervalued (4.5%), lack of intellectual contribution to the research (4.5%), or because of student-perceived lack of ability, skill, or content knowledge (4.3%).

### Finding 4: Students highlighted aspects of the lab environment that contributed to their decisions to stay in research or to consider leaving research

The overall lab environment significantly influenced students’ decisions to stay in their URE and their decisions to leave their URE; notably, the overall lab environment was the only factor that significantly predicted whether students would not consider leaving their URE *and* significantly predicted whether students actually left their URE. Therefore, we chose to explore this particular factor in more depth; we probed what aspects of a lab environment cause students to stay in their URE and what aspects of a lab environment cause students to leave their URE.

We identified all student open-ended responses to questions about why students chose to stay in their URE or why students considered leaving their URE that included the theme of lab environment. We defined a student’s lab environment as a student’s physical, social, and psychological research space, which includes the culture of the lab and the interpersonal relationships that a student may have with other members of the lab, such as postdocs, graduate students, lab staff, and other undergraduate researchers. Using inductive coding, two researchers (K.M.C., and L.E.G.) identified common aspects of positive and negative lab environments within this subset of student responses [55]. The researchers first individually identified themes in student responses. Then, they came together to discuss these themes and reached consensus on a set of common aspects of positive and negative lab environments.

While there is limited research on why undergraduates choose to leave their research experience, studies exploring why people choose to quit their jobs have established “organizational climate,” or one’s work environment, as a critical factor that influences peoples’ decisions to quit their jobs [59]. One study found that fairness, inclusion/exclusion, and social support within one’s organizational environment were key predictors of people’s wellbeing and stress, which ultimately influenced their intention to leave their job [59]. Our analysis revealed that the themes established from students’ open-ended responses echo these findings in the workplace. Thus, we organized the themes into three broader categories: feeling included/excluded, social support/negative social interactions, and fairness in the lab. Below we provide student quotes to support each theme; pseudonyms were given to each of the students and some quotes were lightly edited for clarity.

#### Feeling included/excluded

We identified that students who considered leaving research commonly described a negative lab environment where they felt excluded from the lab. Feelings of inclusion and exclusion exist on a continuum of the degree to which individuals feel they have access to information and resources, feel connected to peers and supervisors, and feel they have the ability to influence decision-making processes [60]. Students such as Timothy and Mia described feeling excluded from the lab.

Timothy (leaver): There was no particular event or person that caused me to leave, I just did not feel like I belonged there.

Mia (leaver): I did not feel like a team member or valued for my presence.

Conversely, students who cited a positive lab environment as a reason why they chose to stay in their URE often described feeling included in the lab. For example, students such as Lauren and Evan described feeling like they were intellectually included as a part of the research team.

Lauren (stayer): I felt like a part of the team and I felt that my thoughts about our research were valued by the grad students/PI; I wasn’t just another data-entering student, I actually contributed to our research.

Evan (stayer): I enjoyed working with the people around me. I felt like I was part of the team and was actively contributing to something greater than myself. I was encouraged to ask questions and never felt like what I asked was dumb.

For students such as Evan and Lauren, feeling a sense of belonging and inclusion seemed to come simultaneously with making intellectual contributions to the research. However, for Andrew, feeling included seemed to be a critical precursor to him putting in additional effort, which resulted in him becoming more of an intellectual contributor to the lab.

Andrew (stayer): I was just going to do the bare minimum and skate by, but as I started to immerse myself in the lab, I found out how much I belonged. They made me feel like I was worth more than just another person persuaded into doing grunt-work. I was quickly pushed to start learning new skills and even reached a point where I was seen as someone who could give meaningful feedback based on the knowledge that I had acquired thus far.

#### Social support/negative social interactions

Students’ relationships with others in the lab also seemed to contribute to their perception of the lab environment; particularly, the extent that a student felt as though they were socially supported by others. Social support refers to supervisory support as well as support from peers [59]. In the context of a URE, social support may be provided by the PI, graduate students, postdocs, lab staff, or other undergraduate researchers. Students who considered leaving their research experience because of a negative lab environment often described feeling as though they had few people to connect or interact with in a meaningful way. As Shreya described, the people in her lab were not necessarily rude or mean, but she did not feel as though she could talk with them.

Shreya (waverer): I was uncomfortable in the environment because it took months before I felt comfortable enough to talk to graduate students in the lab. No one went out of their way to be rude, but I felt like everyone was less inclined to really talk to the undergrads as there weren’t as many of us. People in my lab are extremely focused on their work and it seems to reflect on their mood very often.

While some students such as Shreya simply described a lack of social support, other students such as Meilin and Maddie described overtly negative interactions with members of their lab.

Meilin (waverer): One of the other undergraduates in the lab was extremely rude and passive aggressive towards me.

Maddie (leaver): It was not a conducive work environment—the lab manager was curt and rude.

Moreover, there were examples of extremely negative lab environments where students described situations in which others in the lab treated them poorly, made inappropriate comments, or harassed them.

Mia (leaver): At the end though I had a very negative (harassing) experience with a fellow employee so that made my experience even worse when I look at it in hindsight.

Lynette (waverer): I have considered leaving my research lab due to my graduate student mentor having made inappropriate comments, treated me negatively, and created a negative lab environment for me.

Even though Lynette’s graduate student created a negative lab environment for her, she ultimately chose to stay because she thought that her PI “*is a great mentor*.” However, other students, such as Roslyn, described their PI as the source of the negative lab environment.

Roslyn (leaver): The lab environment was toxic. PI was rude, yelled at students (both undergraduates + graduate students), and that trickled down to an environment where everyone was angry and walking on eggshells. Not supportive, I never felt I could ask questions without being yelled at.

In stark contrast to students who described negative interactions among lab members, students who cited their lab environment as a reason why they chose to stay in research often described positive interactions among lab members. Specifically, students such as Jada and Theresa described interacting with lab members who were supportive and understanding.

Jada (stayer): The lab environment is very encouraging! They are very understanding and encourage you and support you throughout your research project.

Theresa (stayer): Everyone is really supportive of me even when I make a mess or am just generally incompetent, so those are some things that make me feel like I’ll probably stay here.

Additionally, students described lab members that supported them personally as well as professionally.

Emily (waverer): The people I work with and all of my advisors were the reason I stayed. Everyone (…) cared about my personal growth and development.

Students who cited the lab environment as a reason why they chose to stay in research not only described positive relationships among students in the lab, they also described how some relationships had developed into friendships.

Colby (stayer): I developed close friends in the lab. We created a group social media network to talk about our projects, school, and to socialize. As a commuter, this was very important to me since it’s always hard to make friends with similar interests.

Abigail (waverer): The people in the lab are my best friends and they have helped me through my college experience with advice and their friendship.

#### Fairness in the lab

In addition to reporting feelings of exclusion and a lack of support, students who considered leaving their URE because of a negative lab environment also described instances of unfairness in the lab. Unfairness refers to individuals or groups being treated differentially, and the perception of unfairness can emerge when practices and features of the work environment, such as research labs, are seen to benefit some groups more than others [59]. Specifically, students like Carly described instances of favoritism or unequal treatment of undergraduates in the lab.

Carly (leaver): The PI displayed favoritism to some students and allowed those students to make decisions for others, regardless of the skill of either party.

Some students were able to identify what they perceived to be the cause of unfair treatment, such as Abigail who thought that being the only undergraduate who was not a member of the honors college was causing her to be treated unfairly.

Abigail (waverer): I am the only undergraduate student not in the Honors College. I feel I am at a severe disadvantage. I do not get high priority projects, meet with the PI on a consistent basis, or get attention from graduate students as the other Honors Students do. I am not taken seriously and therefore I am frustrated because I am wasting my time. I have been in the lab longer than any of the honors students.

For Abigail, the unequal treatment she experienced in the lab caused her to consider leaving research because it prevented her from working on high priority projects and receiving support from others in the lab. However, for Caroline, the unequal treatment caused her to feel unwelcome in the lab.

Caroline (leaver): I did not like the dynamic between the [staff] and the students. There was a lot of favoritism that made me feel unwelcome or unwanted in the [lab] over others. If it were handled better, I might have understood the blatant unequal treatment of students in the laboratory.

In sum, we identified that students’ relationships with others in the lab is a critical element of the lab environment that affects their decisions to stay in or leave their UREs. Specifically, students who cited that they chose to stay in their UREs because of a positive lab environment often described feeling welcomed into the lab by others and described a strong social support system. Conversely, students who considered leaving their UREs because of a negative lab environment felt unwelcome in the lab and described negative interactions with others in the lab. At times, these students also described situations of unfairness that contributed to their negative perception of the lab environment, which ultimately caused them to consider leaving their UREs.

## Discussion

In this study, we explored what influences students to stay in, to consider leaving, and to actually leave their first URE. We also examined whether the factors that influenced students’ decision-making vary across students of different demographic groups. Although the literature on UREs highlights almost exclusively positive student outcomes [22], we identified that exactly half of the undergraduates in our study had considered leaving their URE, and over a quarter of all students ultimately left their URE. Thus, despite the overwhelming benefits students are poised to gain when participating in a URE, especially a longer URE, not all students choose to stay in their URE until they graduate.

### Increasing student persistence in undergraduate research experiences

**Fig 2:**
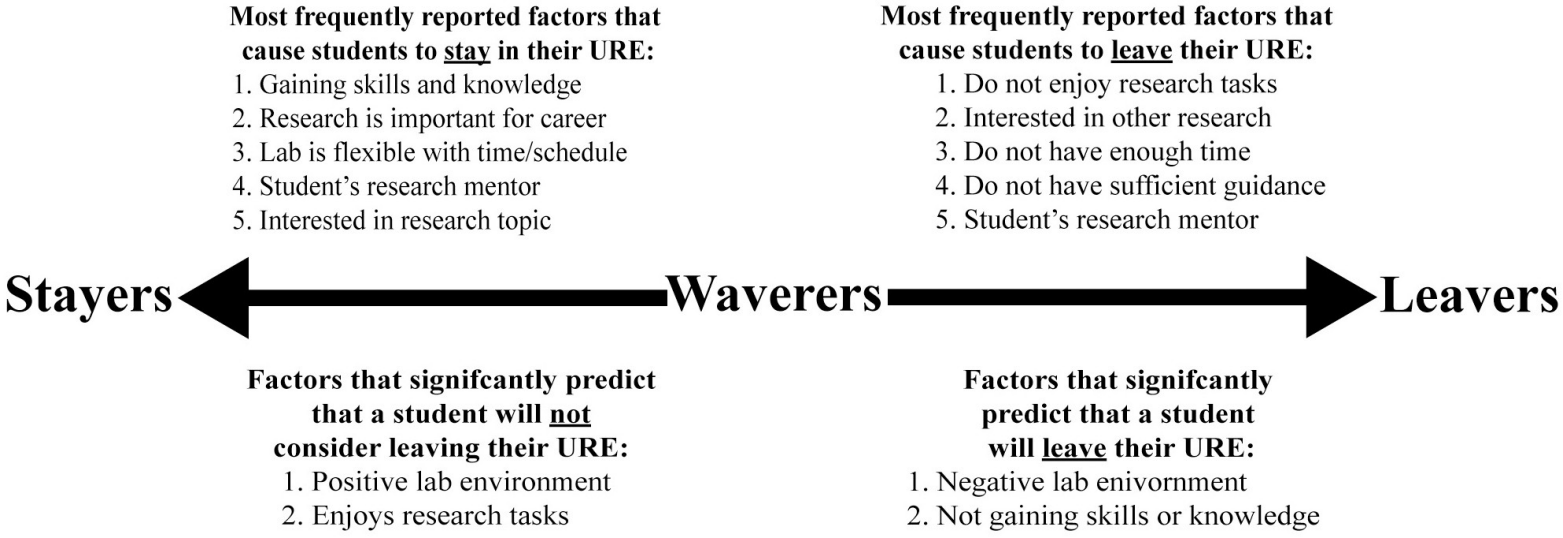
A summary of the primary factors that we identified influence whether students stay in or leave their undergraduate research experience.

Understanding what encourages students to stay in their URE and what causes students to leave their URE provides insight into how mentors can help retain undergraduates in research in hopes of maximizing both their own gains in addition to undergraduates’ gains. Importantly, we recognize that some students leave their research experience to pursue other research opportunities or additional extracurricular activities, which can further enhance their higher education experience and we are not suggesting that efforts should be made to discourage these students from leaving their research experiences. Instead, we intended to identify factors that cause students to leave research that are directly related to their research lab in hopes of identifying concrete ways in which research mentors and others can maximize students’ chances of persisting in these valuable experiences. During this investigation, we were particularly interested in exploring the experiences of waverers in our study, or students who were most vulnerable to leaving because they had considered leaving but ultimately decided to stay. Our data on what causes student to stay and what causes students to leave could be used to try to decrease the number of students who might shift from stayers to waverers and from waverers to leavers. In Fig 2, we highlight the primary factors influencing whether students stay in or leave their URE, including the five most frequently reported factors that cause students to stay in in their URE and the five most frequently reported factors that cause students to leave their URE. We also include the factors that significantly predicted whether a student would not consider leaving their URE and the factors that predicted whether a student would leave their URE.

### What keeps students in their undergraduate research experiences?

We found that students are staying in their URE because they recognize the benefits or value of being involved in research; the two most commonly selected reasons for staying in research were because students perceived they were learning important skills or knowledge and because they perceived it was important for their career goals. This is consistent with literature demonstrating that undergraduate researchers rank discipline-specific knowledge and skills as their primary learning goal in undergraduate research [61]. However, students are also choosing to stay in research because of factors associated with their research labs. Over half of students selected the willingness of their lab to be flexible with their time/schedule, their lab mentor, the overall environment of their lab, and enjoying their everyday research tasks as factors that caused them to stay in their UREs. The importance that students place on a lab’s willingness to be flexible may be particularly important in the life sciences where research can require consistent animal care and where experiments are often time sensitive. In fact, in the open-ended student responses about why students considered leaving research, students expressed frustration with experiments that conflicted with their courses and about having to be in the lab at unusual times and during the holidays.

It is encouraging that students selected their mentor as a key factor influencing their decision to stay in their URE. Research mentors have the opportunity to provide technical, intellectual, and personal/emotional support, as well as professional socialization for students [31,62,63], and studies have demonstrated that positive mentor relationships can lead to an array of benefits for undergraduate researchers [24,54,64,65]. Importantly, we identified that both the lab environment and a student’s enjoyment of research tasks were the two factors that predicted, out of all the students who stayed in their research experience, which ones considered leaving. Our qualitative analyses revealed that feeling welcome in the lab, feeling like a valued member of the research team, and establishing relationships within the lab positively influenced students’ feelings about their lab environment. Thus, research mentors can try to increase the chance that an undergraduate will stay in research by establishing a positive lab environment and encouraging positive relationships among lab members. Additionally, undergraduates valued enjoying their everyday research tasks. Students cited tedious and monotonous tasks as a reason for considering leaving undergraduate research, and also lamented about being asked to do tasks that were too difficult without any guidance. Therefore, to maximize a student’s persistence in research, we suggest ensuring students have a variety of tasks to engage in during their research experience and being attentive to how much guidance a student may need with their research tasks.

The reasons driving students’ decisions to stay in research support the tenets of expectancy value theory, which posits that students must value their experience, believe they will succeed in their research experience, and find little cost to participating in their research experience if they choose to stay [41,42]. Specifically, the two most frequently reported reasons why students chose to stay in their lab environment were directly tied to the value they saw in their research experience: gaining skills and knowledge and research being important for their career goals (Fig 2). Further, value has been shown to be particularly important in decision making. Even if students do not perceive any costs to staying in their research experience, this is likely not sufficient to cause them to stay; they likely have to perceive their research experience as valuable in order to motivate them to stay in the lab [41,42,66].

### Why do students leave their undergraduate research experiences?

Personal reasons, potential opportunities, and aspects of the research lab all factored into why students considered leaving their URE. The five most commonly selected reasons for considering leaving research were that students did not enjoy their research tasks, that they were interested in another opportunity, that they did not have enough time to do research, that they did not have sufficient guidance for their research project, and because of their mentor (Fig 2). It is encouraging that nearly 40% of students indicated that they were interested in leaving because they were more interested in another research opportunity. Although the literature demonstrating the benefits of a longer research experience have primarily focused on the length of a single URE [23,24,26], there is evidence that the number of months a student participates in any combination of research experiences can also lead to increased gains [6]. However, the majority of students did not indicate that they considered leaving because they were interested in another experience. Many students reported leaving because they did not enjoy their everyday lab tasks, which they indicated were often monotonous, tedious, boring, or too difficult for them. Additionally, students considered leaving because of their mentor. Researchers are just beginning to identify examples of negative or problematic mentoring, which can include mismatched work styles, intentional exclusion, and inappropriate delegation, in addition to many other circumstances [67]. Our analysis of students’ open-ended responses echoed these scenarios. In addition to poor mentoring, students also considered leaving research because of absent mentors who provided insufficient guidance. This may explain why in another study, students’ primary suggestion to improve their research experience was increased or more effective faculty guidance [6]. Efforts to improve student/mentor relationships are particularly important because a lack of adequate mentoring has been linked to poor outcomes, such as a loss of interest in graduate school or in the student’s major [37,68]. Finally, students selected that they did not have enough time to do research, and upon further exploration of students’ open-ended responses, we identified that students were often struggling to balance the demands of their courses with the demands of research. This may be particularly true for demographic groups such as community college transfer students, primary caregivers, and low-income students, who likely have competing demands on their time in addition to their course loads [69–72].

We identified that mentors are directly responsible for two of the top five reasons why students consider leaving research and as such, they are well positioned to positively impact a student’s decision to stay in research. Thus, we recommend mentors place high value on their relationships with their undergraduates, paying careful attention to make students feel comfortable, valued, and not overworked. Additionally, mentors should try to be present and available to answer undergraduates’ questions. Notably, research shows that mentor and student perceptions of a mentor’s availability can contradict; although mentors described that they were available to students on a regular and frequent basis, students suggested their meetings were infrequent [73]. Thus, mentors may want to schedule regular meetings with undergraduates [64], in order to ensure that students feel as though they have enough guidance.

The reasons driving students’ decisions to leave research also support the tenets of expectancy value theory [41,42]. Specifically, students saw their current research experience as a cost preventing them from pursuing another research experience, which they presumably valued more. Students also explicitly stated that they did not have enough time to do research (Fig 2). In cases where the value that a student sees in research does not outweigh the costs they perceive of participating in research, they will likely choose to leave their research experience [41,42].

### Working toward a more diverse and inclusive scientific community

The National Academies of Sciences, Engineering, and Medicine cites undergraduate research as a key strategy for broadening participation in STEM and has called for additional efforts to increase the number and the diversity of participants in UREs [22]. As we strive to engage a more diverse group of undergraduates in research, we need to be attentive to how students’ demographics influence their experiences in research so that these students can persist in such opportunities. Below we discuss what influences students of different backgrounds and identities to stay in research and to consider leaving research and how research mentors can provide more inclusive experiences for all students.

#### Gender

We found that males were more likely than females to report staying in research because it is important for their future careers. Using the data we collected from the survey, we explored whether there were gender differences in students’ career goals. Males and females were equally likely to report wanting to pursue a career in research, however males were more likely to want to become medical doctors, while females were more likely to enter other health professions (e.g. nursing, physician assistant) (See the S1 File for these analyses). The value that medical school admissions committees place on participating in undergraduate research [74] may explain why males are more likely to select staying in research because it is important for their careers. We also identified that males are more likely than females to leave their research experience because they do not enjoy their research tasks. This echoes studies demonstrating that men report lower job satisfaction than women despite no observable difference in the quality of job [75]. Researchers suggest this is due to women having lower expectations about what their jobs should entail due to historically worse experiences in the workplace [75]. Finally, we identified that female students were more likely to consider leaving because the lab was not flexible with their schedule. Female students may not be attempting to negotiate more flexible schedules; studies have shown that male college students feel more entitled than their female colleagues to negotiate with instructors [76], which may transfer to their willingness to negotiate with their research mentors. Conversely, this may be because female students have been shown to have more extra-curricular commitments [77] and therefore may have a greater need for a flexible schedule.

These findings suggest that being explicit about the positive impacts of undergraduate research on all career trajectories, regardless of whether research is required for advancement toward a career, may be particularly helpful for female students. Specifically, students may have trouble realizing that the skills they may be gaining in research, such as analytical skills and collaborative skills, are highly valued by employers, many of which do not explicitly list undergraduate research as a required experience [78]. Further, lab mentors should consider being explicit with students about the extent to which they can accommodate scheduling needs (e.g. requiring fewer hours in lab during finals weeks) because female students may be less likely than male students to ask for more flexible schedules.

#### Race/ethnicity

Exploring differences between white and URM students, we found that white students were more likely than URM students to stay in research because they enjoy their everyday lab tasks. Studies have suggested that in predominately white organizations, such as many research institutions, employees of color are expected to work harder on their tasks than white employees to overrule institutional norms [79]. Thus, it is possible that URM students enjoy research less than white students because they either must work harder or do less-desirable tasks, but further research needs to be done to explore this. We also identified that URM students are more likely to consider leaving their research experience because they felt like they were not learning important skills or knowledge. Underrepresented racial minorities have been shown to place greater value on intrinsic work outcomes, such as challenging work and developing new skills [80]. Therefore, this may explain why URM students are more likely than white students to consider leaving research if they do not perceive they are gaining sufficient knowledge and skills.

In order to retain URM students in undergraduate research, we recommend ensuring that students are gaining important knowledge and skills. Mentors can consider backward-designing students’ research experiences by planning specific tasks and activities for undergraduates to do with the intent that they will learn something specific [49]. This may be especially important if the student is working on a part of the project that requires little intellectual engagement.

Exploring differences between white and Asian students, we found that white students were more likely to stay in research because doing research positively contributes to their financial situation. Analyzing demographic data collected from the survey, we found that white students were more likely to report being paid for doing undergraduate research compared to Asian students (See the S1 File for this analysis). While it is difficult to say why this might be, one explanation is that Asian students have been shown to have significantly lower rapport with their faculty research mentors compared to white students [81], which may result in faculty members not mentioning the option of payment to Asian students, or being less willing to pay Asian students. Another possibility is that Asian families sometimes discourage their children from being straightforward and assertive [82], which may result in Asian students being less likely to ask to be paid for their time in the lab. However, more research needs to be done to explore why this gap exists. Additionally, we identified that, compared to white students, Asian students were more likely to stay in research because they are concerned they will not have another opportunity to do research. This may be because Asian students tend to have higher educational expectations than white students [83], and may have a greater fear of the repercussions of leaving and not finding another opportunity. Finally, we identified that Asian students are more likely than white students to consider leaving research because it is not important for their career. Analyzing demographic data from the survey, we found that white students were more likely to report wanting to become scientific researchers than Asian students, but Asian students were more likely to report wanting to become medical doctors. Although research may be an important prerequisite for both careers, it is likely perceived to be most important for becoming a scientific researcher.

An important first step in creating a more equitable research experience for Asian students is to explore why Asian students are less likely than white students to report being paid for doing research. Importantly, this pay gap may partially explain why Asian students are less likely than white students to want to pursue research-related careers [81]. We echo our earlier recommendation to be explicit with all students about how undergraduate research will equip them with skills valuable to many employers and graduate programs, including medical schools. Additionally, we suggest that mentors be as transparent as possible with all students about whether or not they can pay students in exchange for their time in research, and about whether a volunteer research position could eventually result in a paid position.

#### College generation status

Exploring potential differences between first generation college students and continuing generation college students, we found that continuing generation college students were more likely to stay in research because they felt they were learning important skills or knowledge. This may be because first generation college students are less metacognitive about what they are learning [84,85], however this student-deficit explanation places the blame on the first generation students. It is also possible that mentors are less likely to provide first generation students with experiences that provide notable skills and knowledge. Thus, more research needs to be done. We also found that first generation college students were more likely to stay in research because it positively contributes to their financial situation. We identified that first generation students and continuing generation students were equally likely to be paid for their time in research (See the S1 File for this analysis). However, first generation college students are more likely to come from lower socioeconomic backgrounds than continuing generation college students [86,87], and Maslow’s hierarchy of needs would suggest that fulfilling a financial need would be particularly important for these students [44]. Therefore, mentors should consider the potentially positive impact of paying first generation college students for their time in research.

#### Grade Point Average (GPA)

The lower a student’s GPA, the more likely they were to stay in research because they were concerned that they may not have another research opportunity, suggesting that students with lower GPAs likely recognize that securing a URE is competitive and undergraduate researchers tend to have higher-than-average GPAs [6]. We also found that the higher a student’s GPA, the more likely they are to leave their research experience because they do not enjoy their everyday research tasks. Students with lower GPAs may not feel as though they have the option of leaving an unenjoyable experience because they fear they may not obtain a second experience. Additionally, the lower a student’s GPA, the more likely they were to select that they considered leaving research because they did not have enough time to do research. Upon examination of students’ open-ended responses, we found that many students who said they did not have enough time to do research specifically cited that they were struggling to balance the demands of research with the demands of their course load. Thus, it is possible that students with lower GPAs need to put additional time into their academic courses and consequently struggle to find enough time for research.

To both retain academically talented students and to potentially encourage students with lower GPAs to persist in science, it is important to take steps to try to increase the probability that students enjoy their everyday research tasks. Further, we encourage life sciences departments to recognize the academic benefits of undergraduate research in hopes that students can replace core coursework with such experiences, which may help alleviate the struggle students experience balancing research with their course load.

### Application of findings

This research provides unique insight into what causes students to stay in and leave their URE and this information may be of particular interest to people who either advise students to participate in undergraduate research or directly mentor them in undergraduate research. Specifically, research mentors can use these data to inform their own practices; for example, investing more in mentor/mentee relationships and providing sufficient guidance may be particularly helpful in maintaining undergraduate researchers. People who run research programs and work with both research mentors and mentees may want to consider offering workshops that help educate both mentors and mentees about the potential reasons why a student may consider leaving a research experience, highlighting what both parties can do to maximize the student’s time in research. Finally, academic advisors, faculty, or other staff who encourage students to do research and help students find research experiences can maximize a student’s chance of persisting in research by emphasizing that research can be time-intensive. Further, they can highlight to a student how important it can be to find a research experience that is flexible with their time if the student has a constrained schedule, as well as emphasize the potential importance of identifying an area of research that is interesting to the student. However, students should be made aware that not all students get to participate in research [88,89], and that no research experience will be perfect. Thus, they should decide what aspects of a research experience (e.g. schedule flexibility, payment, research area) are most important to them before looking for an opportunity.

### Limitations

This research was limited to public R1 institutions, and the experiences of undergraduate researchers may be different at private colleges and universities, masters granting institutions, primarily undergraduate institutions, and community colleges [53,90]. Our sample also included only life sciences majors, so it is possible that the experiences of undergraduate researchers majoring in other disciplines are different. Additionally, this research relied on student self-report of their experiences in undergraduate research. Some students may have had such negative experiences that they chose not to participate in the survey, or students may have had experiences in research that caused them to switch majors and leave the life sciences. Therefore, we may actually be underestimating students’ negative experiences in undergraduate research. We also asked students to reflect on their *first* URE and collected data at only a single time point. It could have been more difficult for senior students to remember their first URE in detail because it likely would have been longer since their first URE, especially if they chose to leave early in their research experience, and it is possible that students’ memories of their research experiences change over time. Future research taking a longitudinal approach could provide insights into the evolution of student perceptions of research. Additionally, we did not collect data on whether students were required to do research as part of their degree program, although it is likely that most did not. A volunteer effect has been shown for student benefits doing research [91]; if students are required to do undergraduate research, then they may be less likely to try to maximize their experience. If the data presented here primarily comes from students who volunteered to do research, then there is likely a self-selection bias [92] and we do not know how these results may generalize to students who might be required to do undergraduate research. Lastly, our analyses did not account for the intersectionality of student identities [93], which should be explored in future research.

## Conclusion

Half of the undergraduate researchers surveyed considered leaving their first URE. Of students who considered leaving, over half actually left their UREs. Students who reported a positive lab environment or that they enjoy their everyday research tasks were more likely to not consider leaving their URE. In contrast, students who reported a negative lab environment or that they were not gaining important knowledge or skills were more likely to leave their URE. We also identified that student demographics, including gender, race/ethnicity, college generation status, and GPA, significantly predicted which factors influence students to stay in and consider leaving their UREs. Lastly, we identified positive and negative aspects of research lab environments that may critically influence students’ decisions to persist in their undergraduate researcher experience.

## Supporting information

S1 File Supporting information including a copy of the survey, results tables, and additional analyses. (DOCX)

S2 File Anonymized data. (CSV)
