# Supplementary material for: Factors that predict life sciences student persistence in undergraduate research experiences
Source: PLoS One. 2019 Aug 14;14(8):e0220186. doi: 10.1371/journal.pone.0220186 (PMC6693735; doi:10.1371/journal.pone.0220186)
Supplement: S1 File — (DOCX) [file pone.0220186.s001.docx]

Supplemental Information for

***Factors that predict biological sciences student persistence in undergraduate research experiences***

Katelyn M. Cooper^1^*, Logan E. Gin^1^*, Barierane Akeeh^2^, Carolyn E. Clark^2^, Joshua S. Hunter^2^, Travis B. Roderick^2^, Deanna B. Elliott^2^, Luis A. Gutierrez^2^, Rebecca M. Mello^2^, Leilani D. Pfeiffer^2^, Rachel A. Scott^2^, Denisse Arellano^2^, Diana Ramirez^2^, Emma M. Valdez^2^, Cindy Vargas^2^, Kimberly Velarde^2^, Yi Zheng^3^, Sara E. Brownell^1^^

1 The Biology Education Research Lab, Research in Inclusive STEM Education Center, School of Life Sciences, Arizona State University

2 LEAP Scholars, Research for Inclusive STEM Education Center, Arizona State University

3 Mary Lou Fulton Teachers College, Arizona State University

* These authors contributed equally

^Corresponding author. Email: [Sara.Brownell@asu.edu](mailto:Sara.Brownell@asu.edu)

^Corresponding author. Email: [Katelyn.Cooper@asu.edu](mailto:Katelyn.Cooper@asu.edu)

This supplement contains the following:

Item Page

Copy of survey questions analyzed 3

Table S1. Percent of students in the final dataset by institution type and geographic location 6

Table S2. Results of logistic regression testing to what extent student demographics predict 7

whether a student never considers leaving (stays) or considers leaving but stays in

research (wavers).

Table S3. Results of logistic regression testing to what extent student demographics predict 8

whether a student considers leaving research but stays (wavers) or leaves

research (leaves).

Table S4. Additional student demographic information. 9

Table S5. Results of chi square tests of independence testing whether stayers or waverers 10

were more likely to select each of the 11 factors that may have influenced their decision to stay in their first URE.

Table S6.1-11. Results of logistic regressions testing to what extent student demographics 11

predict whether a student checks a particular reason for why they chose to stay in their first URE.

Table S7. Student responses to the open-ended question about why they wanted to stay in 15

their first URE.

Table S8. Results of chi square tests of independence testing whether waverers or 18

leavers were more likely to select each of the 11 factors that may have influenced them to consider leaving their first URE.

Table S9.1-22 Results of logistic regression testing to what extent student demographics predict whether a student checks a particular reason for why they considered leaving their first URE. 19

Table S10. Student responses to the open-ended question about why they considered leaving 23

their first URE.

Table S11. Summary of student-reported career goals 27

Table S12. Results of chi-square tests of independence testing for differences in male and 28

female student intended career goals

Table S13. Results of logistic regression testing to what extent student demographics 29 predict whether a student is paid for their participation in research.

Table S14. Results of chi-squared tests of independence testing for differences in white and 30 Asian student intended career goals

**Copy of survey questions analyzed**

1. Have you, currently or in the past, participated in a scientific undergraduate research experience while enrolled in college? For example, conducting research with a faculty member or in a faculty member's lab.

- Yes
- No *[Students are directed to the end of the survey]*

2. Have you only participated in a summer research experience (e.g., REU) that you did not participate in during the school year?

- Yes *[Students are directed to the end of the survey]*
- No

We are trying to learn more about undergraduate students' experiences in research during the academic school year. During this survey, we would like you to consider your undergraduate research experience. If you have participated in more than one undergraduate research experience, please consider your **FIRST undergraduate research experience** that took place during the academic year when answering the following questions.

4. Have you or did you ever **consider leaving** your **first undergraduate research experience** before graduating from college?

- Yes *[Students are directed to questions 5]*
- No *[Students are directed to question 8 and 9]*

5. Did you **actually leave** your **first undergraduate research experience** before graduating from college?

- Yes, I was asked to leave my research experience *[Students are directed to question 7]*
- Yes, I chose to leave my research experience *[Students are directed to question 6 and 10]*
- No, I did not leave my research experience *[Students are directed to question 6, then 8, then 9, then 10]*

6. Please explain **why you considered leaving** your first research experience. Please be as detailed as possible in your response.

7. Why were you asked to leave your undergraduate research experience? Please be as detailed as possible in your response. *[students are directed to demographic questions]*

8. Please explain **why you chose to stay** in your first research experience. Please be as detailed as possible in your response. *[students are directed to question 9]*

9. Which of the following aspects of your research experience made you **want to stay** in your first research experience? Please select all that apply. If none apply, please go on to the next question.

- Research experience is important for my future career
- Doing research positively contributes to my financial situation
- I have enough time to do research
- I am concerned I may not have another research opportunity
- My mentor who is a PI/faculty member/grad student/post-doc/staff member
- The overall environment of my lab
- The lab is flexible with my schedule/time
- I have sufficient guidance for my research project
- I enjoy my everyday research tasks
- I am interested in my research topic
- I am gaining important skills and knowledge

10. Which of the following aspects of your research experience made you **consider leaving** your first research experience? Please select all that apply. If none apply, please go on to the next question.

- Research experience is not/was not important for my future career
- I need/needed to spend my time making more money than I make/was making doing research
- I do not/did not have enough time to do research
- I am interested/was interested in another research opportunity
- My mentor who is a PI/faculty member/grad student/post-doc/staff member
- The overall environment of my lab
- The lab is not/was not flexible with my schedule/time
- I do not/did not have sufficient guidance for my research project
- I do not/did not enjoy my everyday research tasks
- I am not/was not interested in my research topic
- I am not/was not gaining important skills and knowledge

Demographic questions about research and students

11. Please choose the response that most accurately describes how you are/were compensated for your time working on undergraduate research. **Choose all that apply.**

- I receive/received course credit for my time participating in undergraduate research
- I receive/received money for my time participating in undergraduate research (e.g., wage, stipend)
- I volunteer/volunteered my time in undergraduate research (do not/did not receive credit or money)

12. Please type in the name of the institution you are attending (e.g., Arizona State University, University of Colorado, Boulder)

13. What is your major?

14. I most closely identify as

- Female
- Male
- Other (please describe)
- Decline to state

15. I most closely identify as

- First generation college student whose parents’ highest level of education is a high school diploma or less
- First generation college student (at least one parent has some college)
- Non-first generation college student (at least one parent has finished college)
- Decline to state

16. I most closely identify as

- American Indian or Alaska Native
- Asian
- Black or African American
- Hispanic, Latino, or Spanish origin
- Pacific Islander
- White/Caucasian
- Other (please describe)
- Decline to state

17. What is your grade point average (GPA)?

18. What is your career goal?

19. On average, how many hours per week do/did you spend working on undergraduate research (inside and outside the lab)?

- 1-5 hours
- 6-10 hours
- 11 – 15 hours
- 16 hours or more
- Decline to state

20. How long have you attended college while pursuing your undergraduate degree?

- 1 year or less (first-year student)
- 2 years (sophomore)
- 3 years (junior)
- 4 years (senior)
- 5 years or more
- Decline to state

Table S1. Percent of students in the final dataset by institution type and geographic location

| Institution | n = 768  % (n) |
| --- | --- |
| R1 Public University in the Southwest | 19.0% (146) |
| R1 Public University in the Northeast | 13.4% (103) |
| R1 Public University in the Southwest | 10.5% (81) |
| R1 Public University in the Southwest | 9.4% (72) |
| R1 Public University in the Midwest | 6.1% (47) |
| R1 Public University in the Midwest | 5.5% (42) |
| R1 Public University in the Midwest | 5.1% (39) |
| R1 Public University in the Southeast | 5.1% (39) |
| R1 Public University in the Northwest | 3.0% (23) |
| R1 Public University in the Northeast | 2.9% (22) |
| R1 Public University in the Southeast | 2.6% (20) |
| R1 Public University in the Southeast | 2.6% (20) |
| R1 Public University in the Midwest | 2.2% (17) |
| R1 Public University in the Southeast | 2.0% (15) |
| R1 Public University in the Southwest | 1.7% (13) |
| R1 Public University in the Northeast | 1.6% (12) |
| R1 Public University in the Midwest | 1.4% (11) |
| R1 Public University in the Northeast | 1.3% (10) |
| R1 Public University in the Northwest | 1.3% (10) |
| R1 Public University in the Southwest | 1.2% (9) |
| R1 Public University in the Southeast | 0.8% (6) |
| R1 Public University in the Midwest | 0.5% (4) |
| R1 Public University in the Midwest | 0.5% (4) |
| R1 Public University in the Northwest | 0.3% (2) |
| R1 Public University in the Southeast | 0.1% (1) |

*After excluding students who did not provide complete data on demographic variables, our sample size for regression analyses comparing stayers and waverers is 479, and 375 for comparing waverers and leavers.*

Table S2. Results of logistic regression testing to what extent student demographics predict whether a student never considers leaving (stays) or considers leaving but stays in research (wavers). Reference groups are indicated in parenthesis.

| Model | B ± SE | z value | p-value | Odds ratio |
| --- | --- | --- | --- | --- |
| Intercept | 1.89 ± 1.06 | 1.78 | 0.08 | NA |
| Gender (male) |  |  |  |  |
| Female | -0.38± 0.24 | -1.63 | 0.10 | 1.46 |
| Race (white) |  |  |  |  |
| Asian | 0.25 ± 0.23 | 1.09 | 0.28 | 1.28 |
| URM | 0.70± 0.34 | 2.03 | 0.04 | 2.01 |
| Generation status (non first-gen) |  |  |  |  |
| first generation college student | 0.21 ± 0.24 | 0.87 | 0.39 | 1.23 |
| GPA | -0.28 ± 0.28 | -1.00 | 0.32 | 1.32 |

Table S3. Results of logistic regression testing to what extent student demographics predict whether a student considers leaving research but stays (wavers) or leaves research (leaves). Reference groups are indicated in parenthesis.

| Model | B ± SE | z value | p-value |  |
| --- | --- | --- | --- | --- |
| Intercept | -0.69 ± 1.07 | -0.64 | 0.52 | NA |
| Gender (male) |  |  |  |  |
| Female | -0.15± 0.27 | -0.56 | 0.58 | 1.16 |
| Race (white) |  |  |  |  |
| Asian | 0.09 ± 0.26 | 0.34 | 0.73 | 1.09 |
| URM | 0.58± 0.38 | 1.51 | 0.13 | 1.79 |
| Generation status (non first-gen) |  |  |  |  |
| first generation college student | 0.21 ± 0.26 | 0.80 | 0.42 | 1.23 |
| GPA | 0.24 ± 0.29 | 0.83 | 0.41 | 1.27 |

Table S4. Additional student demographic information of student participants.

|  | **Stayers**  **n = 768**  **% (n)** |
| --- | --- |
| **Demographics** |  |
| Year in college |  |
| First year | 3.8% (29) |
| Second year | 21.8% (148) |
| Third year | 29.8% (229) |
| Four year | 39.7% (305) |
| Fifth year or more | 7.7% (52) |
| Average number of hours spent in research per week |  |
| 1-5 | 18.4% (141) |
| 6-10 | 51.2% (393) |
| 11-15 | 22.1% (170) |
| 16+ | 8.3% (64) |

Table S5. Results of chi square tests of independence testing whether stayers or waverers were more likely to select each of the 11 factors that may have influenced their decision to stay in their first URE.

| Factor | Percent of stayers who selected the factor  n = 384 | Percent of waverers who selected the factor  n = 172 | df | ᵡ^2^ | p-value |
| --- | --- | --- | --- | --- | --- |
| Learning important skills or knowledge | 88.3% | 76.2% | 1 | 13.34 | <0.001 |
| Research is important for career | 84.6% | 82.6% | 1 | 0.38 | 0.54 |
| Lab is flexible with time/schedule | 84.1% | 77.3% | 1 | 3.71 | 0.054 |
| Mentor | 81.8% | 68.6% | 1 | 11.88 | <0.001 |
| Interested in research topic | 79.2% | 60.5% | 1 | 21.27 | <0.001 |
| Lab environment | 78.1% | 48.3% | 1 | 49.45 | <0.001 |
| Enjoys research tasks | 66.1% | 37.2% | 1 | 40.63 | <0.001 |
| Has sufficient guidance | 52.6% | 34.3% | 1 | 15.98 | <0.001 |
| Has enough time for research | 46.6% | 33.7% | 1 | 8.08 | 0.004 |
| Concerned they may not have another opportunity | 20.6% | 42.4% | 1 | 28.60 | <0.001 |
| Research positively contributes to finances | 17.7% | 15.7% | 1 | 0.34 | 0.56 |

Table S6.1. Results table for logistic regression testing to what extent student demographics predict whether a student checks “**I am gaining important skills or knowledge”** as a reason for why they chose to stay in their first undergraduate research experience.

| Model | B ± SE | z value | p-value | Odds ratio |
| --- | --- | --- | --- | --- |
| Intercept | 2.63 ± 1.30 | 2.02 | 0.04 | NA |
| Gender (male) |  |  |  |  |
| Female | 0.01 ± 0.29 | 0.03 | 0.98 | 1.01 |
| Race (white) |  |  |  |  |
| Asian | -0.18 ± 0.30 | -0.60 | 0.55 | 1.20 |
| URM | -0.18 ± 0.38 | -0.48 | 0.63 | 1.20 |
| Generation status (non first-gen) |  |  |  |  |
| first generation college student | -0.66 ± 0.28 | -2.34 | 0.02 | 1.93 |
| GPA | -0.16 ± 0.34 | -0.48 | 0.63 | 1.17 |

Table S6.2. Results table for logistic regression testing to what extent student demographics predict whether a student checks “**Research experience is important for my future career”** as a reason for why they chose to stay in their first undergraduate research experience.

| Model | B ± SE | z value | p-value | Odds ratio |
| --- | --- | --- | --- | --- |
| Intercept | 4.22 ± 1.39 | 3.04 | 0.002 | NA |
| Gender (male) |  |  |  |  |
| Female | -0.65 ± 0.33 | -1.99 | 0.05 | 1.92 |
| Race (white) |  |  |  |  |
| Asian | 0.11 ± 0.30 | 0.37 | 0.71 | 1.12 |
| URM | 0.08 ± 0.40 | 0.21 | 0.83 | 1.08 |
| Generation status (non first-gen) |  |  |  |  |
| first generation college student | -0.37 ± 0.29 | -1.30 | 0.19 | 1.45 |
| GPA | -0.55 ± 0.36 | -1.51 | 0.13 | 1.73 |

Table S6.3. Results table for logistic regression testing to what extent student demographics predict whether a student checks “**My lab was flexible with my schedule/time”** as a reason for why they chose to stay in their first undergraduate research experience.

| Model | B ± SE | z value | p-value | Odds ratio |
| --- | --- | --- | --- | --- |
| Intercept | 1.64 ± 1.20 | 1.37 | 0.17 |  |
| Gender (male) |  |  |  |  |
| Female | -0.13± 0.28 | -0.46 | 0.64 | 1.14 |
| Race (white) |  |  |  |  |
| Asian | -0.31 ± 0.28 | -1.12 | 0.26 | 1.36 |
| URM | -0.38± 0.36 | -1.06 | 0.29 | 1.46 |
| Generation status (non first-gen) |  |  |  |  |
| first generation college student | 0.21 ± 0.28 | 0.73 | 0.46 | 1.23 |
| GPA | 0.03 ± 0.32 | 0.08 | 0.93 | 1.03 |

Table S6.4. Results table for logistic regression testing to what extent student demographics predict whether a student checks “**My mentor who is a PI, faculty member, post-doc, graduate student, or staff member”** as a reason for why they chose to stay in their first undergraduate research experience.

| Model | B ± SE | z value | p-value | Odds ratio |
| --- | --- | --- | --- | --- |
| Intercept | -0.00± 1.07 | -0.00 | 0.10 | NA |
| Gender (male) |  |  |  |  |
| Female | 0.15 ± 0.25 | 0.61 | 0.54 | 1.16 |
| Race (white) |  |  |  |  |
| Asian | -0.24 ± 0.26 | -0.94 | 0.35 | 1.27 |
| URM | -0.62± 0.32 | -1.92 | 0.054 | 1.89 |
| Generation status (non first-gen) |  |  |  |  |
| first generation college student | 0.34 ± 0.26 | 1.28 | 0.20 | 1.40 |
| GPA | 0.35 ± 0.28 | 1.23 | 0.22 | 1.41 |

Table S6.5. Results table for logistic regression testing to what extent student demographics predict whether a student checks “**I was interested in my research topic”** as a reason for why they chose to stay in their first undergraduate research experience.

| Model | B ± SE | z value | p-value | Odds ratio |
| --- | --- | --- | --- | --- |
| Intercept | 0.09 ± 1.02 | 0.09 | 0.93 | NA |
| Gender (male) |  |  |  |  |
| Female | -0.25 ± 0.25 | -1.03 | 0.31 | 1.28 |
| Race (white) |  |  |  |  |
| Asian | -0.19 ± 0.24 | -0.80 | 0.43 | 1.21 |
| URM | -0.08 ± 0.32 | -0.26 | 0.80 | 1.08 |
| Generation status (non first-gen) |  |  |  |  |
| first generation college student | 0.17 ± 0.24 | 0.69 | 0.49 | 1.19 |
| GPA | 0.32 ± 0.27 | 1.20 | 0.23 | 1.38 |

Table S6.6. Results table for logistic regression testing to what extent student demographics predict whether a student checks “**The overall environment of my lab”** as a reason for why they chose to stay in their first undergraduate research experience.

| Model | B ± SE | z value | p-value | Odds ratio |
| --- | --- | --- | --- | --- |
| Intercept | 1.17 ± 0.99 | 1.18 | 0.24 | NA |
| Gender (male) |  |  |  |  |
| Female | -0.42 ± 0.24 | -1.77 | 0.08 | 1.52 |
| Race (white) |  |  |  |  |
| Asian | 0.45 ± 0.24 | 1.85 | 0.06 | 1.57 |
| URM | 0.09 ± 0.30 | 0.30 | 0.76 | 1.09 |
| Generation status (non first-gen) |  |  |  |  |
| first generation college student | -0.32 ± 0.23 | -1.42 | 0.15 | 0.32 |
| GPA | -0.02 ± 0.26 | -0.09 | 0.93 | 1.38 |

Table S6.7. Results table for logistic regression testing to what extent student demographics predict whether a student checks “**I enjoyed my everyday research tasks”** as a reason for why they chose to stay in their first undergraduate research experience.

| Model | B ± SE | z value | p-value | Odds ratio |
| --- | --- | --- | --- | --- |
| Intercept | 0.69 ± 0.94 | 0.74 | 0.46 | NA |
| Gender (male) |  |  |  |  |
| Female | -0.19 ± 0.22 | -0.88 | 0.38 | 1.21 |
| Race (white) |  |  |  |  |
| Asian | -0.22 ± 0.22 | -1.02 | 0.31 | 1.25 |
| URM | -0.75 ± 0.29 | -2.60 | 0.009 | 2.21 |
| Generation status (non first-gen) |  |  |  |  |
| first generation college student | 0.11 ± 0.22 | 0.51 | 0.61 | 1.12 |
| GPA | -0.03 ± 0.25 | -0.11 | 0.91 | 1.03 |

Table S6.8. Results table for logistic regression testing to what extent student demographics predict whether a student checks “**I had sufficient guidance for my research project”** as a reason for why they chose to stay in their first undergraduate research experience.

| Model | B ± SE | z value | p-value | Odds ratio |
| --- | --- | --- | --- | --- |
| Intercept | -1.07 ± 0.95 | -1.12 | 0.26 | NA |
| Gender (male) |  |  |  |  |
| Female | -0.05 ± 0.21 | -0.22 | 0.83 | 1.05 |
| Race (white) |  |  |  |  |
| Asian | -0.06 ± 0.21 | -0.27 | 0.80 | 1.06 |
| URM | -0.56 ± 0.29 | -1.91 | 0.06 | 1.75 |
| Generation status (non first-gen) |  |  |  |  |
| first generation college student | -0.17 ± 0.21 | -0.82 | 0.41 | 1.19 |
| GPA | 0.33 ± 0.25 | 1.29 | 0.20 | 1.39 |

Table S6.9. Results table for logistic regression testing to what extent student demographics predict whether a student checks “**I have enough time to do research”** as a reason for why they chose to stay in their first undergraduate research experience.

| Model | B ± SE | z value | p-value | Odds ratio |
| --- | --- | --- | --- | --- |
| Intercept | -0.48 ± 0.93 | -0.52 | 0.61 | NA |
| Gender (male) |  |  |  |  |
| Female | -0.39 ± 0.21 | -1.85 | 0.06 | 1.48 |
| Race (white) |  |  |  |  |
| Asian | 0.09 ± 0.21 | 0.41 | 0.68 | 1.09 |
| URM | 0.14 ± 0.29 | 0.50 | 0.62 | 1.15 |
| Generation status (non first-gen) |  |  |  |  |
| first generation college student | -0.02 ± 0.21 | -0.08 | 0.94 | 1.02 |
| GPA | 0.13 ± 0.25 | 0.51 | 0.61 | 1.14 |

Table S6.10**.** Results table for logistic regression testing to what extent student demographics predict whether a student checks “**I am concerned I may not have another opportunity”** as a reason for why they chose to stay in their first undergraduate research experience.

| Model | B ± SE | z value | p-value | Odds ratio |
| --- | --- | --- | --- | --- |
| Intercept | 0.54 ± 1.02 | 0.53 | 0.60 | NA |
| Gender (male) |  |  |  |  |
| Female | 0.24 ± 0.25 | 0.98 | 0.33 | 1.72 |
| Race (white) |  |  |  |  |
| Asian | 0.61 ± 0.24 | 2.58 | 0.009 | 1.84 |
| URM | 0.37 ± 0.31 | 1.18 | 0.24 | 1.45 |
| Generation status (non first-gen) |  |  |  |  |
| first generation college student | 0.10 ± 0.24 | 0.43 | 0.67 | 1.11 |
| GPA | -0.56 ± 0.27 | -2.07 | 0.04 | 1.75 |

Table S6.11. Results table for logistic regression testing to what extent student demographics predict whether a student checks “**Doing research positively contributes to my financial situation”** as a reason for why they chose to stay in their first undergraduate research experience.

| Model | B ± SE | z value | p-value | Odds ratio |
| --- | --- | --- | --- | --- |
| Intercept | 0.15 ± 1.14 | 0.14 | 0.89 | NA |
| Gender (male) |  |  |  |  |
| Female | 0.01 ± 0.28 | 0.03 | 0.98 | 1.01 |
| Race (white) |  |  |  |  |
| Asian | -0.82 ± 0.33 | -2.52 | 0.01 | 2.27 |
| URM | -0.40 ± 0.37 | -1.10 | 0.27 | 1.49 |
| Generation status (non first-gen) |  |  |  |  |
| first generation college student | 0.63 ± 0.27 | 2.33 | 0.02 | 1.88 |
| GPA | -0.48 ± 0.30 | -1.60 | 0.11 | 1.61 |

Table S7. Student responses to the open-ended question about why they wanted to stay in their first URE. Significant differences between the percent of students who never considered leaving and the percent of students who considered leaving that reported each theme are marked with an asterisk: * *p* ≤ 0.05 ***p* ≤ 0.01, ****p* ≤ 0.001. Codes that were reported by >5% of students are included.

| Reason students chose to stay in their research experience | Description | All students | Students who never considered leaving  (stayers) | | Students who considered leaving but stayed  (waverers) | |
| --- | --- | --- | --- | --- | --- | --- |
|  |  | % (n)  n=547 | % (n)  n=376 | Student Quote | % (n) n=171 | Student Quote |
| Broadly enjoys the research experience | Student stayed in research because they enjoy the research. This category includes responses describing that the student broadly enjoys the research experience, as well as responses that indicate that the student is interested in their research topic, likes their lab work, or enjoys doing a specific technique. | 41.7% (228) | 48.7% (183)*** | “I was really interested in the work we were doing.” | 26.3% (45) | “I love the work that we do. [I] am genuinely interested in the results/projects.” |
| Positive lab environment | Student stayed in research because they perceive a positive lab environment, have developed positive relationships with or think positively of others in the lab besides their mentor, such as other undergrads or graduate students. | 40.8% (223) | 47.1% (177)*** | “I also got to know the other graduate and undergraduate students in the lab and felt like a part of the community.” | 26.9% (46) | “The people in the lab are my best friends and they have helped me through my college experience with advice and friendship.” |
| Positive relationship with mentor | Student stayed in research because they have developed a positive relationship with their research mentor or that they think positively of their research mentor. A student's PI or anyone whom a student indicated they worked for (e.g. their grad student or post-doc) was considered a mentor. | 27.6% (151) | 32.7% (123)*** | “My mentor cares for me not only as a research assistant but as a person.” | 16.4% (28) | “I have built a strong relationship with my PI.” |
| Opportunity to learn | Student stayed in research because they are already learning or have the opportunity to learn something (e.g. content, skills) from their experience. | 20.8% (114) | 24.7% (93)*** | “I felt that there were many things I could learn and practice.” | 12.2% (21) | “I was learning important things about the research.” |
| Career benefit | Student stayed in research because research is important for their future career, or post-graduate plans. Student indicates that research is important for medical or graduate school, that they are doing research to clarify their career goals, or that they need a letter of recommendation. | 17.9% (98) | 18.1%  (68) | “I am a pre-med so it is one of the important things that I have to do when I apply for medical school so it is very important for me to do research.” | 17.5% (30) | “I am currently staying because I require research lab experience for graduate school.” |
| Receives sufficient help or guidance | Student stayed in research because someone in the lab is available to help them with their research, answer their questions, or to assist them if they need help, guidance, or direction. | 13.3% (73) | 16.5% (62)** | “The other students/faculty were extremely helpful. In a time where I was unclear of a procedure or a detail, I was helped tremendously by other people also working in the lab.” | 6.4%  (11) | “My PI helped me walk slowly through all the parts of the process. She helped me understand all the chemistry that was relevant to our experiments, including the chemistry behind the molecular processes in the cell.” |
| Has independence | Student stayed in their research experience because they have the opportunity to work independently. Specifically, a student can describe that they work on their own research project, have asked their own research question, or feel that they have ownership over a particular project. | 9.0%  (49) | 9.6%  (36) | “My professor also allowed me to take on my own project and control it whatever way I saw fit.” | 7.6%  (13) | “I was also able to make some of my own decisions and use my best judgment on certain projects.” |
| Personal growth or development in research | Student stayed in their research experience because they their research provides them with increased responsibilities, or opportunities for leadership or personal growth and development. | 8.6%  (47) | 8.5%  (32) | “I felt that as I progressed throughout my college career, I'd be allowed to take on more responsibilities in the lab because they professor and other students got to know me better.” | 8.8%  (15) | “I thought/still think it's an incredible growth experience for me.” |
| Commitment and follow through | Student stayed in their research experience because they have a sense of commitment to their research or their research project. Specifically, wanting to see a research project through, wanting to make more progress on their research, or that there is more research to be done. | 7.7%  (42) | 6.6%  (25) | “I started a project which I wanted to see through to the end.” | 9.9%  (17) | “I would have felt bad because the other students who worked there stayed until graduation.” |
| Academic benefit | Student stayed in their research experience because that they want to achieve a specific academic benefit, such as doing honors thesis, receiving a grade, or receiving credit. | 6.4%  (35) | 5.6%  (21) | “I am a graduating senior, and this research constitutes the final credit toward my degree.” | 8.2%  (14) | “I had to stay to complete my honors thesis and graduate with honors.” |
| Lab is accommodating | Student stayed in their research experience because they appreciate how accommodating the lab is of their schedule. Specifically, students often describe how the lab, or their mentor, allows them to work when they want, allows them to work from different locations, or that the lab is accommodating of personal or academic demands on their time. | 6.0%  (33) | 6.6%  (25) | “It was a research project that allowed me flexibility in my schedule and ability to work from home.” | 4.7%  (8) | “My lab is really nice about being understanding of my schedule as a student and off all the other time commitments I have.” |
| Research product | Student stayed in their research experience because they want a research product, such as a published paper or manuscript. This category also includes students who indicate that they want to present their research, or present a poster. | 4.8%  (26) | 4.5%  (17) | “My PI provides me with many opportunities to attend conferences and write manuscripts.” | 5.3%  (9) | “I also realized that the longer I am with a lab, the more likely that I could be published as an undergraduate.” |

Table S8. Results of chi square tests of independence testing whether waverers or leavers were more likely to select each of the 11 factors that may have influenced them to consider leaving their first URE.

| Factor | Percent of waverers who selected the factor  n = 172 | Percent of leavers who selected the factor  n = 204 | df | ᵡ^2^ | p-value |
| --- | --- | --- | --- | --- | --- |
| Did not enjoy lab tasks | 38.4% | 48.0% | 1 | 3.55 | 0.06 |
| Interested in another opportunity | 38.4% | 40.2% | 1 | 0.13 | 0.72 |
| Not enough time | 41.9% | 34.8% | 1 | 1.97 | 0.16 |
| Insufficient guidance | 27.3% | 35.8% | 1 | 3.07 | 0.08 |
| Mentor | 25.6% | 34.3% | 1 | 3.37 | 0.07 |
| Not interested in research topic | 23.8% | 33.3% | 1 | 4.09 | 0.04 |
| Need to make money | 26.7% | 27.0% | 1 | 0.00 | 1.00 |
| Lab environment | 18.0% | 33.8% | 1 | 11.93 | <0.001 |
| Not gaining important skills or knowledge | 11.0% | 25.5% | 1 | 12.71 | <0.001 |
| Research not important for career | 10.5% | 17.2% | 1 | 3.45 | 0.06 |
| Lab not flexible with time or schedule | 11.0% | 11.8% | 1 | 0.05 | 0.83 |

Table S9.1. Results table for logistic regression testing to what extent student demographics predict whether a student checks “**I did not enjoy my everyday research tasks”** as a reason for why they considered leaving their first undergraduate research experience.

| Model | B ± SE | z value | p-value | Odds ratio |
| --- | --- | --- | --- | --- |
| Intercept | -3.00 ± 1.22 | -2.45 | 0.01 | NA |
| Gender (male) |  |  |  |  |
| Female | -0.54 ± 0.27 | -1.98 | 0.05 | 1.72 |
| Race (white) |  |  |  |  |
| Asian | 0.30 ± 0.26 | 1.14 | 0.26 | 1.35 |
| URM | 0.59 ± 0.38 | 1.56 | 0.58 | 1.80 |
| Generation status (non first-gen) |  |  |  |  |
| first generation college student | -0.15 ± 0.27 | -0.56 | 0.58 | 1.16 |
| GPA* | 0.84 ± 0.33 | 2.57 | 0.01 | 2.32 |

Table S9.2. Results table for logistic regression testing to what extent student demographics predict whether a student checks “**I was interested in another research opportunity”** as a reason for why they considered leaving their first undergraduate research experience.

| Model | B ± SE | z value | p-value | Odds ratio |
| --- | --- | --- | --- | --- |
| Intercept | -1.87 ± 1.18 | -1.58 | 0.11 | NA |
| Gender (male) |  |  |  |  |
| Female | 0.28 ± 0.28 | 1.02 | 0.31 | 1.32 |
| Race (white) |  |  |  |  |
| Asian | 0.04 ± 0.26 | 0.16 | 0.88 | 1.04 |
| URM | -0.44 ± 0.40 | -1.11 | 0.27 | 1.55 |
| Generation status (non first-gen) |  |  |  |  |
| first generation college student | -0.48 ± 0.27 | -1.74 | 0.08 | 1.61 |
| GPA | 0.40 ± 0.32 | 1.27 | 0.21 | 1.49 |

Table S9.3. Results table for logistic regression testing to what extent student demographics predict whether a student checks “**I did not have enough time to do research”** as a reason for why they considered leaving their first undergraduate research experience.

| Model | B ± SE | z value | p-value | Odds ratio |
| --- | --- | --- | --- | --- |
| Intercept | 1.94 ± 1.12 | 1.73 | 0.08 | NA |
| Gender (male) |  |  |  |  |
| Female | 0.24 ± 0.28 | 0.86 | 0.39 | 1.27 |
| Race (white) |  |  |  |  |
| Asian | 0.37 ± 0.27 | 1.41 | 0.16 | 1.45 |
| URM | -0.38 ± 0.40 | -0.96 | 0.34 | 1.46 |
| Generation status (non first-gen) |  |  |  |  |
| first generation college student | -0.22 ± 0.27 | -0.80 | 0.42 | 1.25 |
| GPA | -0.73 ± 0.30 | -2.42 | 0.02 | 2.08 |

Table S9.4. Results table for logistic regression testing to what extent student demographics predict whether a student checks “**I did not have sufficient guidance for my research project”** as a reason for why they considered leaving their first undergraduate research experience.

| Model | B ± SE | z value | p-value | Odds ratio |
| --- | --- | --- | --- | --- |
| Intercept | -1.14 ± 1.19 | -0.96 | 0.34 | NA |
| Gender (male) |  |  |  |  |
| Female | -0.13 ± 0.29 | -0.46 | 0.65 | 1.14 |
| Race (white) |  |  |  |  |
| Asian | -0.34 ± 0.29 | -1.16 | 0.25 | 1.40 |
| URM | 0.03 ± 0.39 | 0.08 | 0.94 | 1.03 |
| Generation status (non first-gen) |  |  |  |  |
| first generation college student | -0.13 ± 0.29 | -0.44 | 0.66 | 1.14 |
| GPA | 0.15 ± 0.32 | 0.48 | 0.63 | 1.16 |

Table S9.5. Results table for logistic regression testing to what extent student demographics predict whether a student checks “**My mentor who is a PI, faculty member, graduate student, or staff member”** as a reason for why they considered leaving their first undergraduate research experience.

| Model | B ± SE | z value | p-value | Odds ratio |
| --- | --- | --- | --- | --- |
| Intercept | -2.98 ± 1.33 | -2.24 | 0.03 | NA |
| Gender (male) |  |  |  |  |
| Female | -0.21 ± 0.29 | -0.74 | 0.46 | 1.24 |
| Race (white) |  |  |  |  |
| Asian | 0.12 ± 0.28 | 0.42 | 0.67 | 1.13 |
| URM | -0.34 ± 0.44 | -0.77 | 0.44 | 1.40 |
| Generation status (non first-gen) |  |  |  |  |
| first generation college student | -0.06 ± 0.29 | 0.20 | 0.84 | 1.06 |
| GPA | 0.63 ± 0.36 | 1.76 | 0.08 | 1.88 |

Table S9.6. Results table for logistic regression testing to what extent student demographics predict whether a student checks “**I was not interested in my research topic”** as a reason for why they considered leaving their first undergraduate research experience.

| Model | B ± SE | z value | p-value | Odds ratio |
| --- | --- | --- | --- | --- |
| Intercept | -2.10 ± 1.29 | -1.63 | 0.10 | NA |
| Gender (male) |  |  |  |  |
| Female | -0.05 ± 0.30 | -0.18 | 0.86 | 1.05 |
| Race (white) |  |  |  |  |
| Asian | -0.22 ± 0.30 | -0.74 | 0.46 | 1.25 |
| URM | 0.29 ± 0.40 | 0.73 | 0.47 | 1.34 |
| Generation status (non first-gen) |  |  |  |  |
| first generation college student | -0.37 ± 0.30 | -1.22 | 0.22 | 1.45 |
| GPA | 0.37 ± 0.34 | 1.08 | 0.28 | 1.45 |

Table S9.7. Results table for logistic regression testing to what extent student demographics predict whether a student checks “**I needed to spend time making more money than I made doing research”** as a reason for why they considered leaving their first undergraduate research experience.

| Model | B ± SE | z value | p-value | Odds ratio |
| --- | --- | --- | --- | --- |
| Intercept | 0.18 ± 1.17 | 0.15 | 0.88 | NA |
| Gender (male) |  |  |  |  |
| Female | 0.07 ± 0.31 | 0.23 | 0.82 | 1.07 |
| Race (white) |  |  |  |  |
| Asian | -0.58 ± 0.31 | -1.85 | 0.07 | 1.79 |
| URM | -0.56 ± 0.43 | -1.29 | 0.20 | 1.75 |
| Generation status (non first-gen) |  |  |  |  |
| first generation college student | 0.45 ± 0.29 | 1.57 | 0.12 | 1.57 |
| GPA | -0.33 ± 0.31 | -1.06 | 0.29 | 1.39 |

Table S9.8. Results table for logistic regression testing to what extent student demographics predict whether a student checks “**The overall environment of the lab”** as a reason for why they considered leaving their first undergraduate research experience.

| Model | B ± SE | z value | p-value | Odds ratio |
| --- | --- | --- | --- | --- |
| Intercept | -1.49 ± 1.25 | -1.19 | 0.23 | NA |
| Gender (male) |  |  |  |  |
| Female | -0.52 ± 0.29 | -1.80 | 0.07 | 1.68 |
| Race (white) |  |  |  |  |
| Asian | 0.06 ± 0.29 | 0.19 | 0.85 | 1.06 |
| URM | 0.24 ± 0.41 | 0.59 | 0.55 | 1.27 |
| Generation status (non first-gen) |  |  |  |  |
| first generation college student | 0.09 ± 0.29 | 0.31 | 0.75 | 1.09 |
| GPA | 0.23 ± 0.33 | 0.68 | 0.50 | 1.26 |

Table S9.9. Results table for logistic regression testing to what extent student demographics predict whether a student checks “**I was not gaining important skills or knowledge”** as a reason for why they considered leaving their first undergraduate research experience.

| Model | B ± SE | z value | p-value | Odds ratio |
| --- | --- | --- | --- | --- |
| Intercept | -1.91 ± 1.35 | -1.42 | 0.16 | NA |
| Gender (male) |  |  |  |  |
| Female | 0.15 ± 0.36 | 0.42 | 0.68 | 1.16 |
| Race (white) |  |  |  |  |
| Asian | 0.42 ± 0.33 | 1.28 | 0.20 | 1.52 |
| URM | 0.96 ± 0.42 | 2.29 | 0.02 | 2.61 |
| Generation status (non first-gen) |  |  |  |  |
| first generation college student | -0.09 ± 0.34 | -0.28 | 0.78 | 1.09 |
| GPA | 0.03 ± 0.36 | 0.07 | 0.94 | 1.03 |

Table S9.10. Results table for logistic regression testing to what extent student demographics predict whether a student checks “**Research experience was not important for my future career”** as a reason for why they considered leaving their first undergraduate research experience.

| Model | B ± SE | z value | p-value | Odds ratio |
| --- | --- | --- | --- | --- |
| Intercept | -3.91 ± 1.81 | -2.16 | 0.03 | NA |
| Gender (male) |  |  |  |  |
| Female | 0.38 ± 0.45 | 0.84 | 0.40 | 1.46 |
| Race (white) |  |  |  |  |
| Asian | 0.77 ± 0.37 | 2.06 | 0.04 | 2.16 |
| URM | 0.70 ± 0.53 | 1.34 | 0.18 | 2.01 |
| Generation status (non first-gen) |  |  |  |  |
| first generation college student | -0.33 ± 0.42 | -0.79 | 0.43 | 1.39 |
| GPA | 0.39 ± 0.48 | 0.81 | 0.42 | 1.48 |

Table S9.11. Results table for logistic regression testing to what extent student demographics predict whether a student checks “**The lab was not flexible with my schedule/time”** as a reason for why they considered leaving their first undergraduate research experience.

| Model | B ± SE | z value | p-value | Odds ratio |
| --- | --- | --- | --- | --- |
| Intercept | -0.94 ± 1.54 | -0.61 | 0.54 | NA |
| Gender (male) |  |  |  |  |
| Female | 1.39 ± 0.62 | 2.23 | 0.03 | 4.01 |
| Race (white) |  |  |  |  |
| Asian | 0.44 ± 0.39 | 1.12 | 0.27 | 1.55 |
| URM | -0.65 ± 0.68 | -0.96 | 0.34 | 1.91 |
| Generation status (non first-gen) |  |  |  |  |
| first generation college student | -0.05 ± 0.42 | -0.12 | 0.91 | 1.05 |
| GPA | -0.66 ± 0.39 | -1.68 | 0.09 | 1.93 |

Table S10. Student responses to the open-ended question about why they considered leaving their first URE. Significant differences between the percent of students who considered leaving but stayed and the percent of students that left research that reported each theme are marked with an asterisk: * *p* ≤ 0.05 ***p* ≤ 0.01, ****p* ≤ 0.001. Codes that were reported by >5% of students are included.

| Reasons why students considered leaving their research experience | Description | All students | Students who left  (leavers) | | Students who considered leaving but stayed  (waverers) | |
| --- | --- | --- | --- | --- | --- | --- |
|  |  | % (n)  n=375 | % (n)  n=204 | Student Quote | % (n) n=171 | Student Quote |
| Broadly disinterested in/does not enjoy research | Student considered leaving or left research because they did not enjoy the research topic, the topic is not of interest to them, or they find the research boring. The student may also describe that the research doesn’t align with their major or academic interests. | 21.1%  (79) | 22.1%  (45) | “I decided to leave my first research experience primarily due to lack of interest in the research.” | 19.9% (34) | “I was not very interested in the topic of research of the lab.” |
| Personal time constraints | Student considered leaving or left research because working in the lab interferes with their personal commitments, such as academics or time in their personal schedule. | 17.6%  (66) | 15.7%  (32) | “There was too much going on with my school and work schedule.” | 19.9% (34) | “I was working a part time job and taking full time classes along with research and my grades began to suffer as a result.” |
| Insufficient guidance and/or absent mentor | Student considered leaving or left research because their mentor is absent or that they do not have sufficient guidance for their research. | 12.8%  (48) | 15.7%  (32) | “I did not have any idea what I was doing and was provided no guidance from the faculty member whose research it was.” | 9.4% (16) | “My mentor provided me with very little training. I felt as if she had no time to meet with me or provide hands-on guidance. (…) Not once during this time did she check my procedures or results.” |
| Intention to seek a different research opportunity | Student considered leaving or left research to seek or explore another research lab or opportunity, but have not explicitly stated that they have transitioned to a different opportunity. | 10.1%  (38) | 5.9% (12) | “I wanted to get some research experience in a different department.” | 15.2% (26)** | “I considered leaving my first and only undergraduate lab experience so I could gain experience in other labs doing different things.” |
| Negative PI/Mentor Relationship | Student considered leaving or left their research experience because they have a negative relationship with their mentor. A student's PI or anyone whom the student indicated they worked for is considered their mentor. | 9.3%  (35) | 9.8%  (20) | “The supervisor would constantly berate me and the other undergrads about how we were not good enough and were lazy (we were not).” | 8.8%  (15) | “The mentor was very condescending and not pleasant to work with.” |
| Negative lab environment | Student considered leaving or left their research experience because of a negative lab environment or because they have a negative relationship with others in the lab besides their mentor. | 8.5%  (32) | 9.8%  (20) | “The overall culture of the lab wasn't very inviting.” | 7.0%  (12) | “One of the other undergraduates in the lab was extremely rude and passive aggressive towards me.” |
| More interested in another area | Student considered leaving or left their research because they find another area more interesting than what they are doing. | 7.7%  (29) | 9.3%  (19) | “The research experience was as a tech in a fish lab feeding fish and cleaning tanks, so once I found a new research opportunity more in line with my interests (medical research) I left my first opportunity for that one.” | 5.8%  (10) | “I considered leaving just because I became more interested in infectious disease research and microbiology than the metabolomics research we were doing.” |
| Work is tedious or monotonous | Student considered leaving or left their research because they felt the lab work they are doing is boring, repetitive, tedious, or monotonous. Additionally, a student could describe their work as not challenging enough or that they would rather be doing something else. | 6.9%  (26) | 6.9%  (14) | “I hated working in the lab (...) The work was boring and hard on the eyes (it involved staring at a computer screen the entire time) and monotonous.” | 7.0%  (12) | “My job was to do a literature review and that also was terribly uninteresting.” |
| Found different/better research opportunity | Student considered leaving or left their research because they have found, transitioned to, or are actively participating in another research lab or research opportunity. | 6.9%  (26) | 10.3%  (21)** | “I left the first lab because there was another research opportunity that was more relevant to my interests. “ | 2.9%  (5) | “I started another research experience and was not confident I could juggle two different research experiences along with classes.” |
| Lack of personal growth or benefit | Student considered leaving or left their research because their lab did not offer opportunities for advancement in lab tasks, promotion, and responsibilities. Additionally, a student can describe that they did not feel they were growing as a scientist. | 6.1%  (23) | 7.4%  (15) | “I did not think that I would grow as a scientist in that lab.” | 4.7%  (8) | “I wanted to get lab experience, but it didn't seem worth my time if I wasn't gaining anything from the experience.” |
| Lack of structure/  disorganized lab | Student considered leaving or left their research experience because the lab is lacking structure or because the lab or their mentor is disorganized. | 4.5%  (17) | 7.4%  (15)** | “The lab was extremely chaotically run. The P.I. didn't even know all the students that worked in his lab- years after I left it, I still periodically get emails from him.” | 1.2%  (2) | “The lab was extremely disorganized.” |
| High time commitment | Student considered leaving or left their research experience because the research requires a lot of time or too much time. | 4.5%  (17) | 2.9%  (6) | “The work became too time-consuming.” | 6.4%  (11) | “It is also a lot of hours per week to dedicate to the lab.” |
| Feels overworked or undervalued | Student considered leaving or left their research experience because they were overworked or not properly compensated for the time they spent in lab. Additionally, students could mention that they did not feel as though they were valued in the lab. | 4.5%  (17) | 5.4%  (11) | “Lab techs were paying me to be a lab assistant for 15 hrs/week but expected me to come in for additional time to do research.” | 3.5%  (6) | “I sometimes felt overworked as an unpaid undergraduate student.” |
| Lack of learning | Student considered leaving or left their research because expected to learn, or are not learning, information or lab techniques in their lab. | 4.5%  (17) | 5.4%  (11) | “I felt that I was not learning as much as I could [have been].” | 3.5%  (6) | “I felt like I would never learn anything.” |
| Lack of intellectual contribution to project | Student considered leaving or left their research because they do not feel like they are contributing intellectually to a project or participating in meaningful discussions. Additionally, students can describe that the tasks that they are doing are menial or not important to advancing the research project. | 4.5%  (17) | 5.4%  (11) | “I didn't get to participate in projects or conversations about addressing problems in the research.” | 3.5%  (6) | “When I considered leaving the research experience is when it felt like I was doing grunt work and not really taking part in the experiment that was meaningful.” |
| Student realizes their lack of ability, skill, or content knowledge | Student considered leaving or left their research because they feel as though they struggle in their research experience due to a lack of content knowledge, lack of ability, or lack of skills. Additionally, they can describe that they have difficulties meeting expectations or keeping up in the lab. | 4.3%  (16) | 5.8%  (6) | “I was totally lost when I was researching about the topic. I didn't know many terms and it was really hard for me to understand the research papers I was reading that time.” | 2.9%  (10) | “During the early phase of my time at the research lab I was given a computer coding task that I could not complete. I spent several months trying but was not skilled enough to overcome the obstacles.” |

Table S11. Summary of student-reported career goals

|  | Total |
| --- | --- |
|  | n = 786  % (n) |
| ^a^Medical doctor | 44.8% (344) |
| ^b^Scientific researcher | 27.1% (208) |
| cOther health professional | 15.9% (122) |
| ^d^Other science career | 3.5% (27) |
| ^e^Other | 3.1% (24) |
| ^f^Unsure | 4.7% (36) |
| Decline to state | 0.9% (7) |
| Students responded to an open-ended question asking, “What is your career goal?” Responses were coded into the following categories:  ^a^Medical doctor: includes careers as doctors or some specialty within medicine (e.g., cardiologist, pediatrician)  ^b^Scientific researcher: includes research or academic careers, such as professor or biologist  ^c^Other health professional: includes healthcare-related careers but not medical doctor (e.g., nursing, physician’s assistant, physical therapy)  ^d^Other science career: includes careers that is science-related but not healthcare or research (e.g., industry, biotechnology)  ^e^Other: includes careers that are not science, research, or healthcare related (e.g., law, education)  ^f^Unsure: includes students who said they did not know what they wanted to do or were undecided with regards to their career goal | |

Table S12. Results of chi-squared tests of independence testing for differences in male and female student intended career goals

|  | Male  n = 186 | Female  n = 568 | df | ᵡ^2^ | p-value |
| --- | --- | --- | --- | --- | --- |
| Medical doctor | 51.1% | 42.6% | 1 | 4.07 | 0.04 |
| Scientific researcher | 29.6% | 26.2% | 1 | 0.79 | 0.37 |
| Other health professional | 9.7% | 18.1% | 1 | 7.44 | 0.006 |
| Other science career | 1.1% | 4.2% | 1 | 4.18 | 0.04 |
| Other | 2.7% | 3.3% | 1 | 0.20 | 0.66 |
| Unsure | 5.4% | 4.4% | 1 | 0.30 | 0.58 |
| Decline to state | 0.5% | 1.1% | NA | NA | NA |

Table S13. Results of logistic regression testing to what extent student demographics 28 predict whether a student is paid for their participation in research.

| Model | B ± SE | z value | p-value | Odds ratio |
| --- | --- | --- | --- | --- |
| Intercept | -0.50 ± 0.89 | -0.57 | 0.57 |  |
| Gender (male) |  |  |  |  |
| Female | 0.07 ± 0.22 | 0.35 | 0.73 | 1.07 |
| Race (white) |  |  |  |  |
| Asian** | -0.78 ± 0.24 | -3.23 | 0.001 | 2.18 |
| URM | 0.21 ± 0.26 | 0.80 | 0.42 | 1.23 |
| Generation status (non first-gen) |  |  |  |  |
| first generation college student | 0.10 ± 0.21 | 0.57 | 0.64 | 1.11 |
| GPA | -0.16 ± 0.23 | -0.70 | 0.48 | 1.17 |

Table S14. Results of chi-squared tests of independence testing for differences in white and Asian student intended career goals

|  | White  n = 416 | Asian  n = 204 | df | ᵡ^2^ | p-value |
| --- | --- | --- | --- | --- | --- |
| Medical doctor | 37.7% | 54.9% | 1 | 16.41 | <0.001 |
| Scientific researcher | 33.2% | 16.6% | 1 | 18.60 | <0.001 |
| Other health professional | 15.1% | 19.6% | 1 | 1.97 | 0.16 |
| Other science career | 4.3% | 2.0% | 1 | 2.21 | 0.14 |
| Other | 3.8% | 2.0% | 1 | 1.56 | 0.21 |
| Unsure | 5.0% | 3.9% | 1 | 0.39 | 0.53 |
| Decline to state | 0.7% | 1.0% | NA | NA | NA |
